# FMRP, FXR1 protein and *Dlg4* mRNA, which are associated with fragile X syndrome, are involved in the ubiquitin–proteasome system

**DOI:** 10.1038/s41598-023-29152-4

**Published:** 2023-02-02

**Authors:** Hideo Shimizu, Hirohiko Hohjoh

**Affiliations:** Department of Molecular Pharmacology, National Institute of Neuroscience, NCNP 4-1-1 Ogawahigashi, Kodaira, Tokyo, 187-8502 Japan

**Keywords:** Proteasome, Ubiquitylation, Neurogenesis, Neurological disorders

## Abstract

The ubiquitin–proteasome system (UPS) is a proteolytic pathway that is essential for life maintenance and vital functions, and its disruption causes serious impairments, *e.g.*, disease development. Thus, the UPS is properly regulated. Here we show novel UPS-related factors: the fragile X mental retardation 1 (FMR1) and Fmr1 autosomal homolog 1 (FXR1) proteins and *discs large MAGUK scaffold protein 4* (*Dlg4*) mRNA, which are associated with Fragile X syndrome, are involved in UPS activity. *Fmr1*-, *Fxr1*- and *Dlg4*-knockdown and *Fmr1*- and *Fxr1*-knockdown resulted in increased ubiquitination and proteasome activity, respectively. FXR1 protein was further confirmed to be associated with proteasomes, and *Dlg4* mRNA itself was found to be involved in the UPS. Knockdown of these genes also affected neurite outgrowth. These findings provide new insights into the regulatory mechanism of the UPS and into the interpretation of the pathogenesis of diseases in which these genes are involved as UPS-related factors.

## Introduction

For quality control of proteins in cells, unwanted proteins and poorly synthesized proteins are actively degraded. The ubiquitin–proteasome system (UPS) is the primary ubiquitin-dependent proteolytic pathway. Unwanted proteins are labelled with ubiquitin by the ubiquitination system, in which ATP-dependent E1 ubiquitin-activating enzyme, E2 ubiquitin-conjugating enzyme, and E3 ubiquitin ligase are involved^1,2^. Ubiquitinated proteins are rapidly and selectively degraded by proteasomes. The UPS plays an essential role in various biological functions such as cell cycle progression, cell differentiation, signal transduction as well as intracellular protein homeostasis^1,2^. Thus, abnormalities in the UPS most likely cause serious impairments, for example, disease development such as neurodegenerative diseases^1,3–6^.

Fragile X syndrome (FXS) is an inherited mental and intellectual development disorder and frequently shows autistic spectrum as well^7^. The responsible gene for FXS is the *fragile X mental retardation 1* (*FMR1*) gene on chromosome Xq27.3, and an aberrantly expanded CGG repeat (> 200 repeats) in the 5’ untranslated region (UTR) of *FMR1* causes the deficiency of the FMR1 protein (also called as FMRP), resulting in the onset of FXS^7^. The mutant *FMR1* gene with 50–200 CGG repeats also causes Fragile X-associated tremor/ataxia syndrome (FXTAS), a late-onset neurodegenerative disorder that includes cerebellar ataxia, Parkinsonism, memory and executive dysfunction, autonomic dysfunction and other cognitive declines^7,8^.

*FMR1* and the *FMR1 autosomal homolog 1* (*FXR1*) and 2 (*FXR2*) genes belong to the fragile X-related (*FXR*) gene family^9^. These genes encode RNA-binding proteins that have high sequence similarity in functional domains, *e.g.*, the K homology (KH) and RGG box domains for RNA binding^9^. FMRP is highly expressed in the brain and is thought to play an important role in the translational regulation of several messenger RNAs (mRNAs) that bind to FMRP in postsynaptic neurons, suggesting its contribution to synaptic function such as synaptic plasticity^9^.

The FXR1 protein is ubiquitously expressed and is known to have an important role in the development of cardiac and skeletal muscle^9,10^. FXR1 forms heterodimers with FMRP^11^, and these proteins presumably share a common mRNA target^12^, shuttle between the nucleus and cytoplasm^13^ and associate with polyribosomes^14^. FXR1, like FMRP, may be associated with FXS^9^. In addition, the associations of FXR1 with other neurological disorders such as bipolar disorder and schizophrenia, and with heart and muscle diseases and carcinogenesis have been reported^9,10^. However, the functional mechanism of FXR1 remains poorly understood.

Discs large MAGUK scaffold protein 4 (DLG4: also called as postsynaptic density protein 95, PSD95) is a major scaffold protein in postsynaptic density and plays an important role in synaptic function^15^. *Dlg4* mRNA is known to be transported from the cell body to post-synapses and translated there^16^. FMRP is a carrier of *Dlg4* mRNA, binding to and stabilizing the mRNA and regulating its translation^16^.

Little is known about the relationship between the UPS and the *FXR* and/or *Dlg4* genes, but we have found close relationship among them in this study. Knockdown of *Fmr1*, *Fxr1* and *Dlg4* increased ubiquitination levels, and knockdown of *Fmr1* and *Fxr1* increased proteasome activity. In addition, an association between FXR1 and proteasome was also found. These results suggest that the genes are involved in the UPS. Furthermore, knockdown of these genes was showed to inhibit neurite outgrowth in neuronal cells. This suggests that the UPS may contribute to neurite outgrowth. The findings provide new insights into the regulation of the UPS and the pathogenesis of diseases in which *Fmr1*, *Fxr1* and *Dlg4* are involved as UPS regulators.

## Results

### Reduced GFP expression under *Dlg4*- and *Fxr1*-knockdown conditions

**Figure 1:**
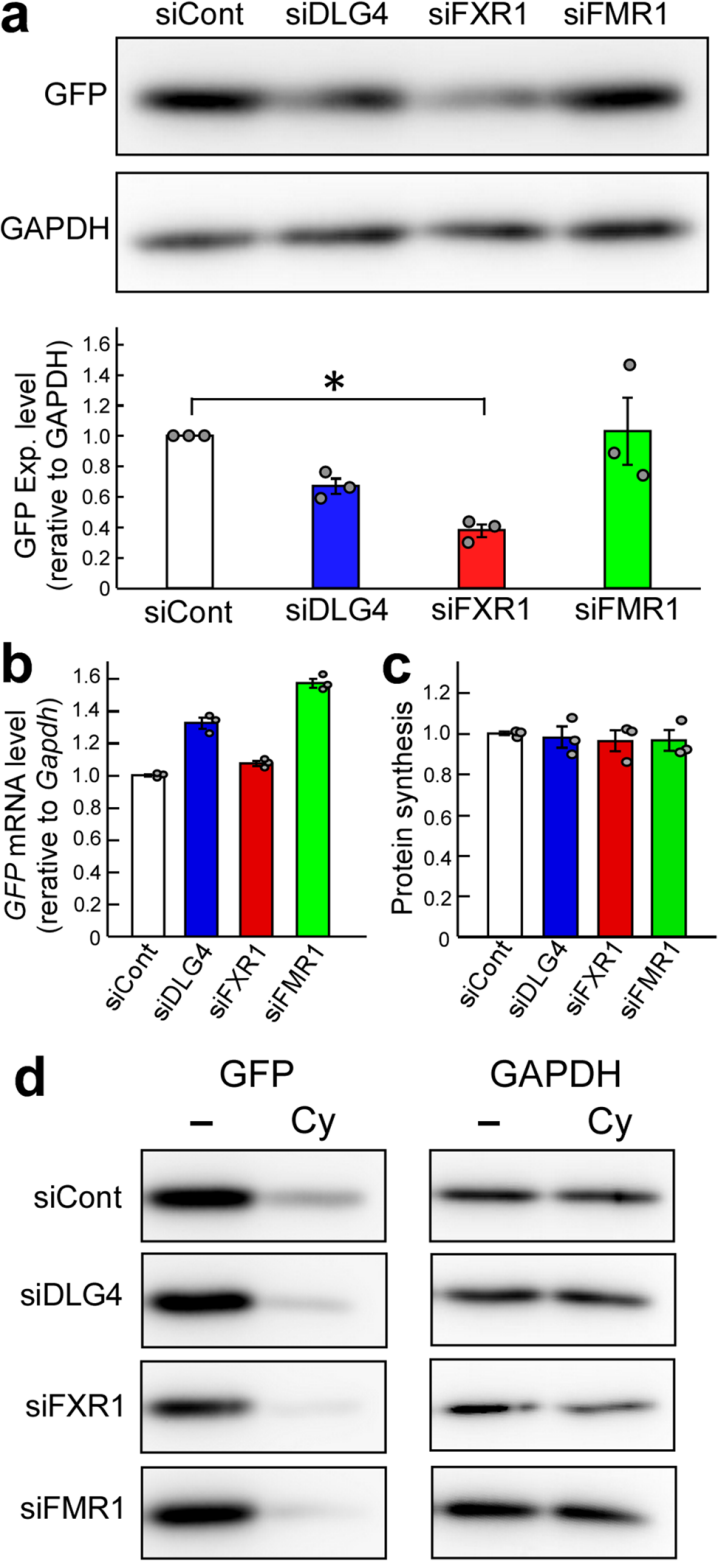
GFP expression under *Dlg4*-, *Fxr1*- and *Fmr1*-knockdown conditions. (**a**) GFP expression. The pd2EGFP-N1 plasmid encoding the *GFP* gene was transfected into N2a cells with the indicated siRNAs. siCont: a non-silencing control siRNA. 48 h after transfection, cell lysate was prepared and divided into two portions for western blotting and qRT-PCR analyses. Protein concentrations were measured, and equal amounts of lysates were examined by western blotting with anti-GFP and GAPDH antibodies. The experiment was triplicated (the other two blots are shown in Supplementary Fig. 1), and signal intensities of GFP and GAPDH bands were measured with the ImageJ software. GFP intensities were normalized by GAPDH intensities and further normalized by the data obtained from cells treated with siCont as 1. Data are shown as mean ± SEM (*n* = 3 examinations, *P < 0.05 by one-way analysis of variance with Dunnett’s post hoc tests). (**b**) *GFP* transcript amount. Total RNAs were extracted from the same N2a cells as in (**a)**, and the *GFP* and *Gapdh* transcripts were examined by qRT-PCR. The data were analyzed by the delta-delta Ct method using the *Gapdh* data as a reference and normalized by the data obtained from cells treated with siCont as 1. Data are shown as mean ± SEM (n = 3 independent determination). (**c**) Protein synthesis level. Overall nascent proteins under gene knockdown conditions (indicated) were examined by a protein synthesis assay kit. Fluorescently labeled nascent proteins were measured and signal intensities were averaged. The data were further normalized by the data obtained from cells treated with siCont. Data are shown as mean ± SEM (n = 3 independent determination). (**d**) Protein stability. Protein stability was examined using cycloheximide as a translation inhibitor. N2a cells transfected as in **(a)** were treated with cycloheximide (Cy) and its vehicle (-) (DMSO) for 9 h and examined by western blotting as in (**a**).

We observed that the green fluorescence protein (GFP), examined as a reporter, was reduced in Neuro2a (N2a) cell, a mouse neuroblastoma cell line, where *Dlg4* and *Fxr1* were knocked down (Fig. 1a and Supplementary Fig. 1). This provided a clue to discovering a novel relationship between the ubiquitin–proteasome system (UPS) and the *FXR* and *Dlg4* genes. In addition, the following points should be noted: N2a cells express *Dlg4* mRNA, but the DLG4 protein is hardly detectable, suggesting that *Dlg4* mRNA is translationally suppressed in the cells (Supplementary Fig. 2).

The *GFP* gene is driven by the CMV promoter in the pd2EGFP-N1 plasmid used. Assuming that gene expression would change depending on the state of N2a cells undergoing gene silencing (knockdown), the *GFP* transcription and protein synthesis (translation) levels were examined by quantitative RT-PCR (qRT-PCR) and using a protein synthesis assay kit that labels and detects overall nascent proteins in the cells, respectively. As a result, sufficient amounts of *GFP* transcripts (Fig. 1b) and similar amounts of labeled nascent proteins (Fig. 1c) were detected among the cells examined, suggesting little difference in transcription and translation levels among the cells.

### Post-translational regulation of GFP

The protein stability of GFP was examined in *Dlg4-* and *Fxr1*-knockdown cells. GFP-expressing N2a cells with *Dlg4-* and *Fxr1*-knockdown were treated with cycloheximide, a protein synthesis inhibitor, and GFP and endogenous GAPDH were examined by western blotting. 9-h cycloheximide treatment reduced the GFP level. But, interestingly, the GFP signal intensity of *Dlg4-* and *Fxr1*-knockdown cells, as well as *Fmr1*-knockdown cells, after cycloheximide treatment was even more reduced compared to that of siCont (non-silencing siRNA) and cycloheximide-treated cells (Fig. 1d). The lowest intensity of GFP was observed in *Fxr1*-knockdown cells after cycloheximide treatment. The signal intensity of endogenous GAPDH also appeared to be reduced in *Fxr1*-knockdown cells with cycloheximide treatment compared to that in siCont-treated cells. These results, together with the above results, suggest that *Dlg4-* and *Fxr1*-knockdown N2a cells possess a lower level of protein stability than intact N2a cells. Thus, *Dlg4* and *Fxr1* may be related to post-translational regulation.

### Increased ubiquitination and proteasome activity by *Dlg4*-, *Fxr1*- and *Fmr1*-knockdown

The UPS is a major pathway that regulates intracellular proteolysis^1,2^. *Dlg4-* and *Fxr1-*knockdown N2a cells, which showed a marked decrease in GFP (Fig. 1a), were treated with MG132, a proteasome inhibitor, and the GFP level was examined. MG132 treatment increased GFP levels, suggesting that proteasome is involved in GFP stability (Supplementary Fig. 3).

**Figure 2:**
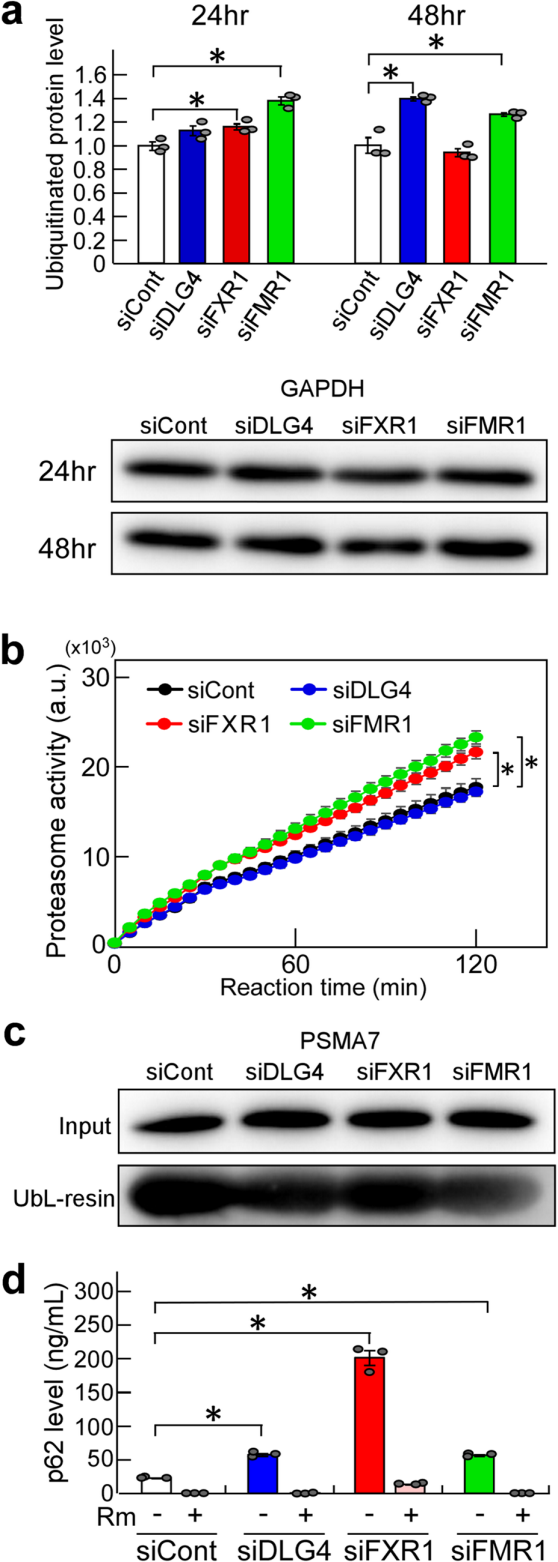
Ubiquitination and proteasome activity. (**a**) Ubiquitinated protein levels. N2a cells were transfected with the indicated siRNAs and incubated for 24 or 48 h. The level of total polyubiquitinated proteins in the cells was measured by ELISA. Briefly, cell extracts were prepared, and protein concentrations were determined. Equal amounts of samples (approximately 1.8 µg/µl) were subjected to testing by ELISA, and further examined by western blotting with anti-GAPDH antibody. The ELISA data were normalized with the data obtained from cells treated with siCont as 1. Data are shown as mean ± SEM (*n* = 3 independent determination, *P < 0.05 by one-way analysis of variance with Dunnett’s post hoc tests). (**b**) Proteasome activity. N2a cells were treated as in (**a)**. After 48-h gene knockdown, cell lysates were prepared, and protein concentrations were measured. Proteasomes were isolated from equal amounts of lysate (approximately 300 µg) by precipitation with UbL- and control-resin, and their activity was examined by an assay kit. The fluorescent signal produced from a fluorescent-labeled substrate by proteasome activity was measured. Data with UbL-resin were subtracted by data with control-resin (background value) and plotted at arbitrary units (a.u.). Data are shown as mean ± SEM (*n* = 3 independent determination, *P < 0.05 by two-way analysis of variance). (**c)** Isolated proteasomes. Isolation of proteasomes was performed as in (**b**). Concentration-adjusted N2a cell lysates for proteasome preparation (input) and isolated proteasomes (UbL-resin) were examined by western blotting for PSMA7, an essential subunit of the 20S proteasome complex. **(d**) P62 levels. N2a cells were subjected to gene knockdown as in **(a**), incubated for 44 h and treated with (+) and without (-) rapamycin (Rm), an inducer of autophagy, for 4 h. Intracellular p62 levels were examined by ELISA. Cell extracts were prepared, and protein concentrations were measured. Equal amounts of samples (approximately 700 ng/µl) were examined by ELISA. Data are shown as mean ± SEM (*n* = 3 independent determination, *P < 0.05 by one-way analysis of variance with Dunnett’s post hoc tests).

We examined whether *Dlg4-*, *Fxr1-* and *Fmr1-*knockdown affects intracellular ubiquitination using an ELISA, which detects and quantifies total intracellular polyubiquitinated proteins. In *Dlg4-*, *Fxr1-* and *Fmr1-*knockdown N2a cells, ubiquitinated protein levels were increased (Fig. 2a). However, 48 h-*Fxr1-*knockdown cells, in contrast to 24 h-*Fxr1-*knockdown cells, showed ubiquitination levels comparable to control cells, which will be discussed later.

Next, we examined the activity of proteasomes isolated from *Dlg4-*, *Fxr1-* and *Fmr1-*knockdown N2a cells. The results were interesting: proteasome activity was significantly increased in *Fxr1-* and *Fmr1-*knockdown cells compared to siCont-treated cells (Fig. 2b). In contrast, the proteasome activity in *Dlg4-*knockdown cells was the same, *i.e.*, unchanged, as in siCont-treated cells.

Ubiquitinated proteins are degraded not only by proteasome, but also by autophagy involving the p62/SQSTM1 (p62) protein, an adaptor that can bind ubiquitinated proteins^17^. In particular, aggregates of ubiquitinated proteins are trapped by p62 and are selectively degraded by autophagy. Thus, the level of p62 was examined in *Dlg4-*, *Fxr1-* and *Fmr1-*knockdown cells, and found to be significantly increased in the cells (Fig. 2d); the highest level was observed in *Fxr1*-knockdown cells. The increased p62 was markedly reduced by treatment with rapamycin (Fig. 2d), an inhibitor of mTOR (mammalian target of rapamycin), which can induce autophagy, suggesting that autophagy was functioning normally in the cell. Therefore, increased ubiquitinated protein aggregates, which are trapped by p62 and subjected to autophagic proteolysis, are thought to accumulate through a rate-limiting proteolytic reaction in autophagy. In any case, the finding is consistent with and supportive of increased ubiquitination by *Dlg4-*, *Fxr1-* and *Fmr1-*knockdown.

As for the observed low level of ubiquitination in 48 h-*Fxr1-*knockdown cells (Fig. 2a), it is possible that p62-bound ubiquitinated protein aggregates which accumulated intracellularly may be difficult to detect with the assay used, while non-aggregated ubiquitinated proteins, which are detectable by the assay, were rapidly degraded by the enhanced proteasome and significantly reduced in the cells. To confirm this possibility, further studies are needed.

In addition to the above findings, as a contrasting result, data on ubiquitination and proteasome activity in *Prnp* (*Prion protein*) and *Atf4* (*Activating transcription factor 4*) knockdown cells were shown to be almost comparable to the data of control cells (Supplementary Fig. 4). These suggest that the increase in ubiquitination and proteasome activity is not triggered by arbitrary gene silencing (knockdown). In other words, there is specificity in gene knockdown that affects the UPS.

When taken together, the findings strongly suggest that *Dlg4-*, *Fxr1-* and *Fmr1-*knockdown affects UPS activity, *i.e.*, *Dlg4*, *Fxr1* and *Fmr1* are involved in the UPS.

### Effects of *Dlg4*- and *Fxr1*-knockdown on UPS activity in the absence of FMRP

The FXR1 protein and *Dlg4* mRNA are known to be associated with FMRP^11,16^. It is of interest whether the effect of *Dlg4-* and *Fxr1-*knockdown on UPS activity is dependent on FMRP. To see the effect of *Dlg4-* and *Fxr1*-knockdown on the UPS in the absence of FMRP, we generated a FMRP-deficient N2a cell line (named def.FMRP-N2a cell) by gene editing with the CRISPR-Cas9 system (Supplementary Fig. 5a); and def.FMRP-N2a cells were subjected to gene silencing for *Dlg4* and *Fxr1* followed by ubiquitination and proteasome activity assays as above. The results indicated that ubiquitination and proteasome activity were increased in the cells with *Dlg4-* and *Fxr1-*knockdown and in *Fxr1-*knockdown cells, respectively (Supplementary Fig. 5b,c), which are consistent with the results using naïve N2a cells (see Fig. 2). Thus, *Dlg4-* and *Fxr1-*knockdown promotes ubiquitination independently of FMRP, and FXR1 is related to proteasome activity independently of FMRP. In addition to the above double-gene suppression conditions, a combination of *Fxr1-* and *Dlg4-*knockdown was also examined. As a result, in both ubiquitination and proteasome assays, the results of double-gene knockdown cells were similar to those of *Fxr1* single-knockdown cells, *i.e.*, there were few additive and synergistic effects in the double-gene knockdown cells (Supplementary Fig. 6). This suggests that *Fxr1*-knockdown may have a greater impact on the UPS than *Dlg4*-knockdown.

### Association between FXR1 protein and *Dlg4* mRNA

**Figure 3:**
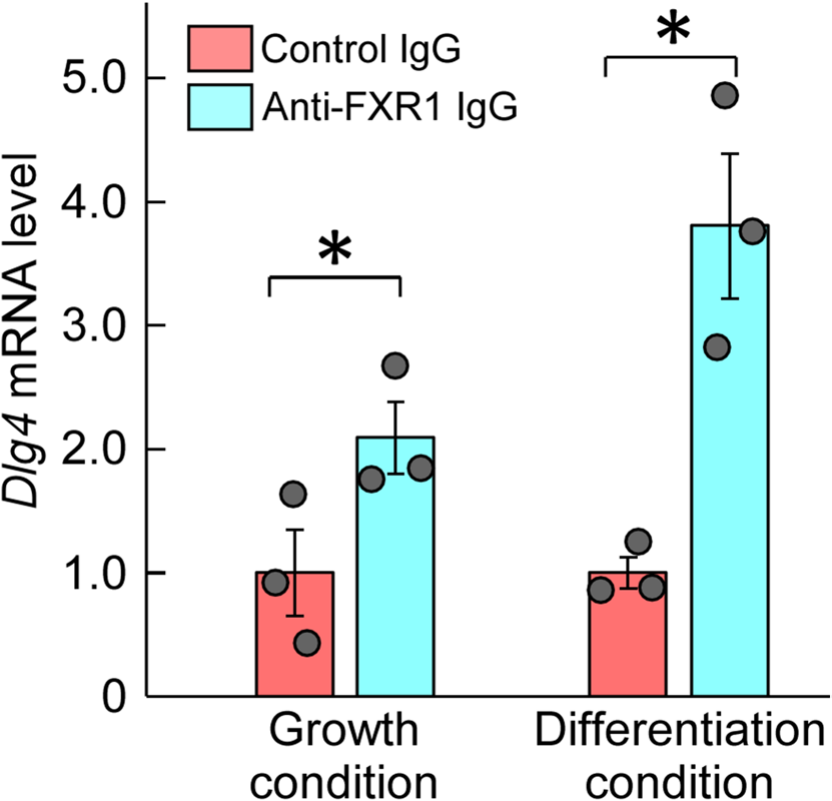
Association between FXR1 protein and *Dlg4* mRNA. Immunoprecipitation with anti-FXR1 antibody was performed in def.FMRP-N2a cells (in the absence of FMRP) cultured under normal growth and retinoic acid (RA)-added differentiation conditions. RNA was extracted from the precipitants with anti-FXR1 IgG or control IgG and examined by qRT-PCR for *Dlg4*. The data were normalized by the data of control IgG as 1. Data are shown as mean ± SEM (*n* = 3 independent determination, *P < 0.05 by one-tailed t-test).

Based on the amino acid sequence homology between FXR1 and FMRP, FXR1 is expected to bind to *Dlg4* mRNA as well as FMRP. The binding of FXR1 to *Dlg4* mRNA was examined in the absence of FMRP effects, *i.e.*, using def.FMRP-N2a cells. RNAs coimmunoprecipitated with anti-FXR1 antibody from def.FMRP-N2a cell extracts were examined by qRT-PCR for *Dlg4*. The results indicated the presence of *Dlg4* mRNA in the precipitants, suggesting that *Dlg4* mRNA is associated with FXR1 protein (Fig. 3).

### FXR1 binds to proteasome, but FMRP does not

**Figure 4:**
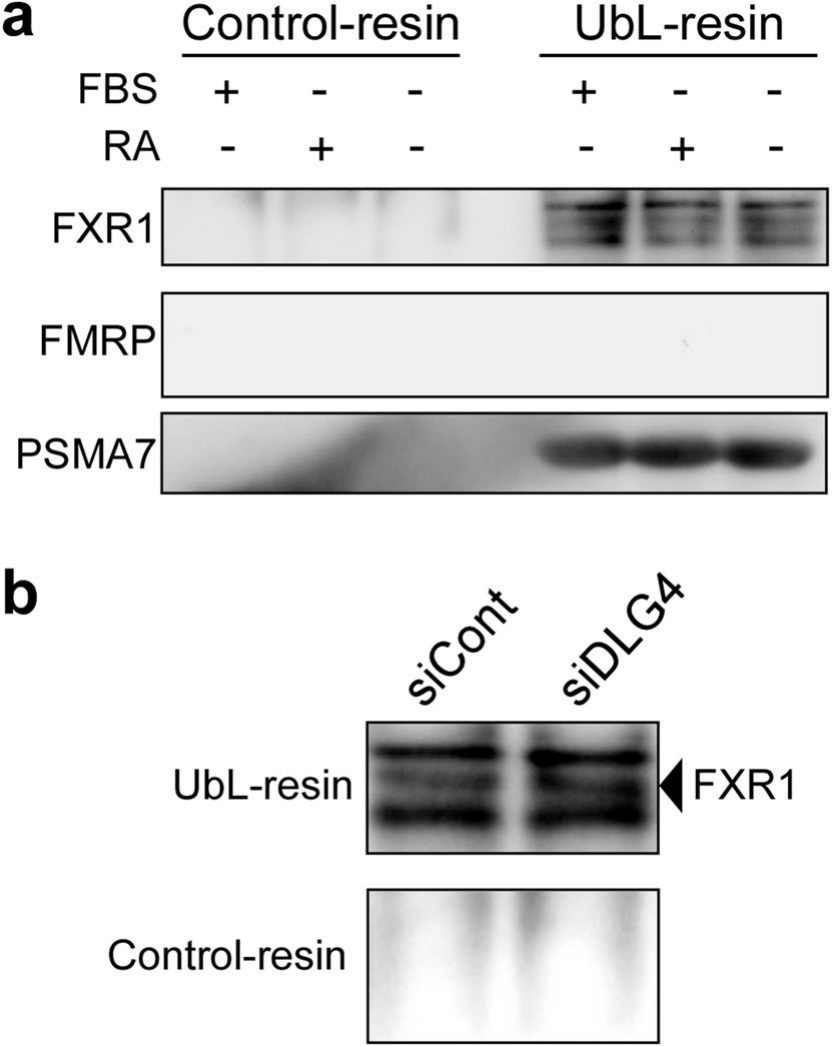
Association of FXR1 protein with proteasome. (**a**) Association between FXR1 and proteasome. Proteasomes were collected by precipitation with the UbL-resin and Control-resin from N2a cells cultured under growth conditions (FBS +) and serum-free differentiation conditions with (FBS-/RA +) or without (FBS-/RA-) retinoic acid. The collected proteasomes were examined by western blotting for FXR1, FMRP and PSMA7 (an essential subunit of the 20S proteasome complex). (**b**) Association of FXR1 with proteasome under *Dlg4*-knockdown conditions. Proteasomes were collected from N2a cells treated with siDLG4 and siCont, and examined by western blotting for FXR1 as in (**a**).

The findings so far raised an important question: do FMRP and FXR1 bind to proteasome? To address the question, we collected proteasomes from cells using the UbL-Resin containing the ubiquitin-like domain and examined them by western blotting for FMRP and FXR1. The results were intriguing: FXR1 was found to be present in the collected proteasomes, but FMRP was not detected (Fig. 4a). This strongly suggests that FXR1 protein binds to proteasomes, but FMRP does not.

We further examined whether FXR1 can bind to proteasomes even under *Dlg4-* knockdown conditions; this is because *Dlg4* mRNA is associated with FXR1 (Fig. 3). The results showed that FXR1 coprecipitated with proteasomes even under *Dlg4-* knockdown conditions (Fig. 4b), suggesting that *Dlg4* mRNA may not mediate FXR1 binding to proteasomes.

### Effects of *Dlg4*-, *Fxr1*- and *Fmr1*-knockdown on process formation and UPS activity under differentiation conditions

**Figure 5:**
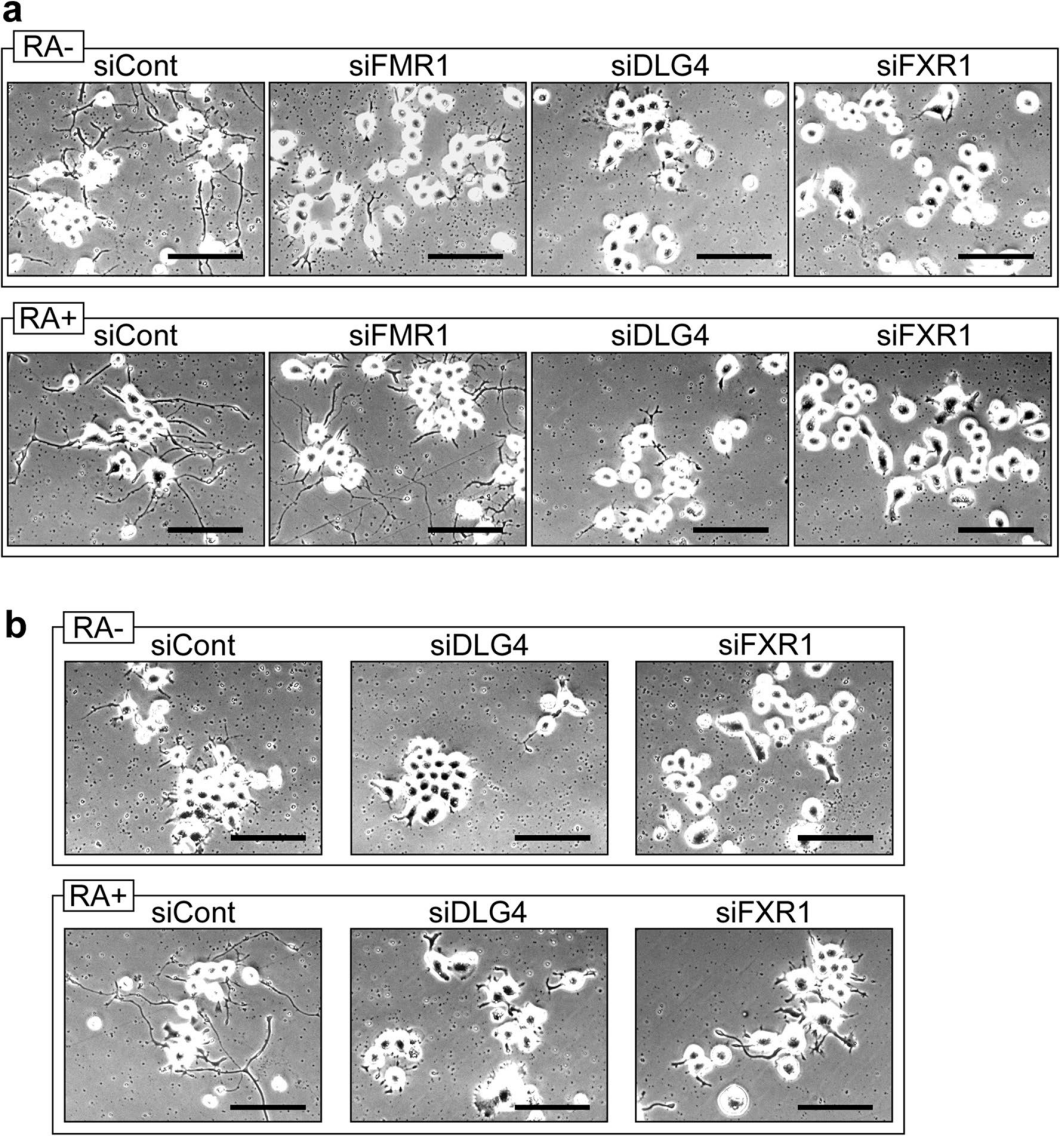
Process formation of N2a cell. (**a**) N2a cells with gene knockdown under differentiation conditions. N2a cells were transfected with the indicated siRNAs and incubated in serum-free differentiation medium with (RA +) or without (RA-) retinoic acid for 3 days. The cells were observed by a microscope. Scale bars indicate 100 μm. (**b**) Def.FMRP-N2a cells with gene knockdown under differentiation conditions. Def.FMRP-N2a cells were treated and observed as in (**a**). Scale bars indicate 100 μm.

N2a cell can form neurite-like processes under serum-free and retinoic acid (RA)-added culture conditions (differentiation conditions)^18^. When *Dlg4*, *Fxr1* and *Fmr1* were knocked down, the resulting N2a cells suppressed process formation under serum-free differentiation conditions (Fig. 5a). However, supplementation with RA restored process formation in *Fmr1*-knockdown N2a cells, but not in *Dlg4-* and *Fxr1*-knockdown cells (Fig. 5a). Similarly, in def.FMRP-N2a cells, process formation occurs under RA-added differentiation conditions but is cancelled by *Dlg4-* and *Fxr1*-knockdown (Fig. 5b).

UPS activity was examined in N2a cells under differentiation conditions. As shown in Supplementary Fig. 7a, ubiquitination was increased, but proteasome activity remained unchanged except for a slight decrease under serum-free differentiation conditions. Similar results were also obtained in def.FMRP-N2a cells under differentiation conditions (Supplementary Fig. 7b).

The effects of *Dlg4-*, *Fxr1*- and *Fmr1*-knockdown on UPS activity under differentiation conditions were examined. Gene knockdown of *Dlg4* and *Fmr1* (for 48 h) increased ubiquitination under differentiation conditions, and *Fxr1*- and *Fmr1*-knockdown significantly increased proteasome activity (Supplementary Fig. 7c,d). These results are consistent with the results of N2a cells under growth culture conditions (Fig. 2). Consequently, the effects of *Dlg4-*, *Fxr1*- and *Fmr1*-knockdown on UPS activity do not appear to change under either growth culture or differentiation conditions.

### Effects of *Dlg4*- and *Fxr1*-knockdown on neurite outgrowth

**Figure 6:**
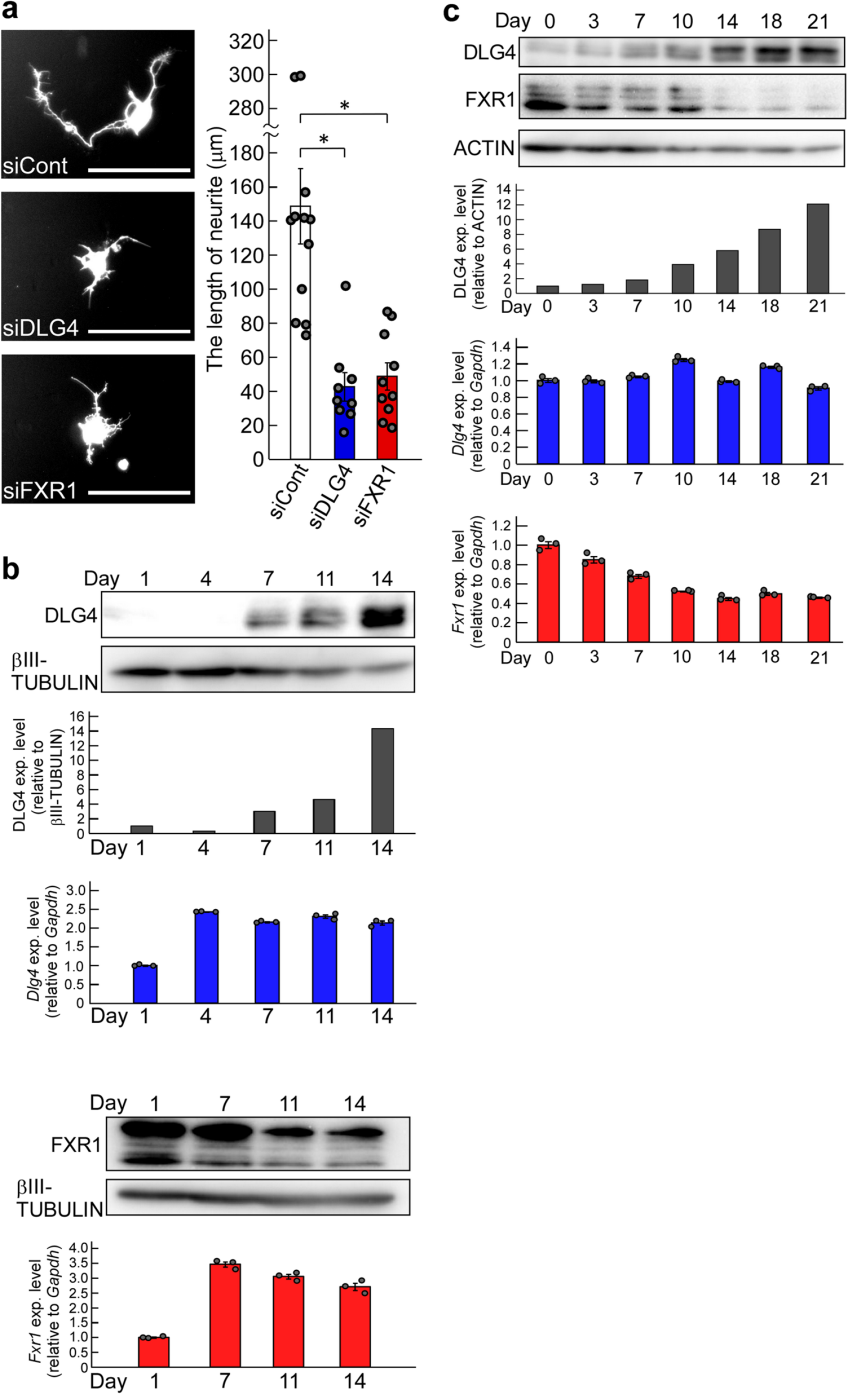
Effects of *Dlg4*- and *Fxr1*-knockdown on neuritogenesis, and the gene expression profiles. (**a**) Effects of *Dlg4*- and *Fxr1*-knockdown on neurite outgrowth. The day (day 0) before transfection, primary mouse neurons were prepared and seeded onto culture plates. Transfection of the indicated siRNAs together with the *GFP* gene as a reporter was performed (day 1). 3 days after transfection (day 4), GFP-positive neurons were examined, and representative neurons were exhibited (see photos). Scale bars indicate 100 μm. The longest neurite of each neuron was measured and at least 9 neurons were examined in each siRNA-transfected neuron (see graph). The data were compared to the data obtained from siCont-transfected neurons (*P < 0.05 by one-way analysis of variance with Dunnett’s post hoc tests). Data are shown as mean ± SEM. (**b**) Expression of *Dlg4* and *Fxr1* during culture of primary neurons. Day 1 is the day after the preparation and seeding of primary neurons as in (**a)**. Proteins and total RNAs were extracted from neurons on the indicated day and examined by western blotting and qRT-PCR, respectively. In western blot, DLG4, FXR1 and βIII-TUBULIN (as a loading control) were examined. Signal intensities of DLG4 and βIII-TUBULIN bands were measured with the ImageJ software. DLG4 intensities were normalized by βIII-TUBULIN intensities and further normalized by Day1 data as 1 (black bar-graph). The qRT-PCR data of *Dlg4* (blue bar-graph) and *Fxr1* (red bar-graph) were analyzed by the delta-delta Ct method using the data of *Gapdh* as a reference and further normalized by the data of Day 1 as 1. Data are shown as mean ± SEM (*n* = 3 independent determination). **(c**) Expression profiles of *Dlg4* and *Fxr1* during postnatal mouse brain development. Proteins and total RNAs were extracted from the brain at the indicated postnatal day (Day 0 is delivery day) and examined as in **(b)**. In western blot, ACTIN was examined as a loading control. DLG4 intensities were normalized by ACTIN intensities and further normalized by Day0 data as 1 (black bar-graph).

The inhibition of process formation in N2a cells with *Dlg4-* and *Fxr1*-knockdown is particularly striking (Fig. 5). To determine whether the gene knockdown also affects actual neurite outgrowth, primary mouse neurons were prepared and transfected with *Dlg4* and *Fxr1* siRNAs together with the *GFP* gene as a reporter; and neurite outgrowth was examined in GFP-positive neurons. As expected, *Dlg4-* and *Fxr1*-knockdown markedly suppressed neurite outgrowth of GFP-positive neurons (Fig. 6a). We further examined the expression of the target genes (*Dlg4* and *Fxr1*) in the cultured neurons. The results of *Dlg4* are noteworthy: early in the culture, when neurite outgrowth is active (see siCont-treated neurons in Fig. 6a), *Dlg4* mRNA was present in sufficient amounts in neurons, but little DLG4 protein was detected (see day 1 and day 4 in Fig. 6b). In contrast, *Fxr1* mRNA and the protein were detected in sufficient amounts throughout the culture period. These results are consistent with those obtained in N2a cells (Supplementary Fig. 2a). Thus, *Dlg4* mRNA is expressed at sufficient levels during neurite outgrowth but is translationally suppressed. This together with the impact of *Dlg4*-knockdown suggest that *Dlg4* mRNA itself has a function in neurons during neurite outgrowth.

Neurite elongation and synapse formation are essential for postnatal brain development. We investigated the expression profiles of *Dlg4* and *Fxr1* over the course of postnatal mouse brain development. As shown in Fig. 6c, sufficient amounts of *Dlg4* mRNA were detected during postnatal brain development, but only small amounts of DLG4 protein were present during the first few days, after which DLG4 protein gradually increased. In contrast, *Fxr1* mRNA and the protein, which correlate with each other, were expressed in sufficient amounts at birth, gradually decreased, and stabilized after postnatal day 14 (Fig. 6c). These results are consistent with the in vitro results (Fig. 6b and Supplementary Fig. 2) and support an important role for *Dlg4* mRNA in neurite outgrowth during postnatal brain development.

## Discussion

The UPS, by which unwanted and poorly synthesized proteins are systematically degraded, is essential for maintaining homeostasis in vivo, and its dysfunction is thought to cause various impairments such as disease development^1,2^. The UPS is properly regulated, and its regulation is complex. For example, various E3 ubiquitin ligases and their substrate specificities represent some of the complexity^2^. Elucidating the regulatory mechanisms of the UPS is vital for understanding homeostatic mechanisms based on protein quality control. In this study, we have shown that the *FXR* and *Dlg4* genes, which are related to Fragile X syndrome (FXS)^9,19^, are associated with the UPS. Knockdown of these genes promote UPS activity (Fig. 2). Furthermore, we have found that FXR1 protein is associated with proteasome (Fig. 4), and that *Dlg4* mRNA, but not the protein, is implicated in the UPS (Fig. 2 and Supplementary Fig. 2). Although the regulatory mechanism of the UPS, such as the substrate specificity of the E3 ubiquitin ligase involved in selective proteolysis^2^, are known about the specificity of UPS, the factors presumed to regulate UPS activity as shown here may be unique.

FMRP is an RNA binding protein that is known to be involved in mRNA transport and localization in neuronal cells and in protein synthesis at the synapse^7,9^. FMRP plays a variety of roles in neuronal cells, but its function has not been fully understood. The deficiency of FMRP causes FXS, to which aberrantly expanded CGG repeat in the 5’ UTR of *Fmr1* is closely related^7,9^. The loss of FMRP does not result in neuronal cell death, but rather in neurodevelopmental abnormalities^20,21^. FMRP-knockdown and -knockout N2a cells are similarly non-lethal, but process formation depends on the presence or absence of retinoic acid in culture conditions. This may reflect the vulnerability of FMRP-deficient cells to neuritogenesis. Deficient FMRP is thought to cause dysregulation of the translation of mRNAs that bind to FMRP, which in turn causes various manifestations of FXS.

This study adds new insights into the interpretations of the pathogenesis of FXS; that is, dysregulation of the UPS due to FMRP deficiency may be involved in the pathogenesis of FXS. Accumulation of ubiquitinated protein aggregates has been observed in the brain of FXTAS^22,23^ and in neurons of *Fmr1*-knockout mice^21^. The observed increase in ubiquitination due to FMRP deficiency in this study (Fig. 2) is consistent with the previous observations. Thus, these suggest that FMRP deficiency affects the UPS.

The remaining issue in understanding the dysregulation of UPS due to FMRP deficiency is that the degradation capability in relation to the accumulation of ubiquitinated proteins needs to be studied extensively, including not only proteasome activity but also autophagy and levels of ubiquitination.

FXR1 has been reported to be associated with various diseases as well as vital functions^9,10^, but its functional mechanism has not yet been fully elucidated. Since FXR1 is an RNA binding protein similar to FMRP^9^, RNA metabolism and translational regulation by RNA binding of FXR1 may be involved in the functional mechanism. In this study, we have found that FXR1 binds to proteasome and that proteasome activity is increased in the absence of FXR1 (Figs. 2, 4), suggesting that FXR1 plays an important role in proteasome regulation. In addition, it also appeared to be involved in intracellular ubiquitination (Fig. 2). Since little was known about the relationship between FXR1 and the UPS, these findings are unexpected and provide us with new insight into the functional mechanism of FXR1. Furthermore, reconsidering FXR1 as a UPS-related factor may provide a new interpretation of the pathogenesis of FXR1-associated diseases. For example, protein quality control problems caused by FXR1 dysfunction may be involved in FXR1-associated diseases. Further progress and studies are expected to elucidate new functional mechanisms of FXR1 as a UPS-related factor in biological functions and pathogenesis in the future.

Another intriguing finding in this study is that the *Dlg4* mRNA is functional and involved in the UPS (Fig. 2 and Supplementary Fig. 2). DLG4 (also called PSD95) is a well-known protein involved in synapse formation and functions^15^. In contrast, little is known about intracellular contribution and function of the *Dlg4* mRNA. Thus, the finding was unexpected and attracted our interest. *Dlg4* mRNA is thought to bind to a variety of proteins including FMRP and FXR1, and one such *Dlg4* mRNA-protein complex may work as a UPS regulator. *Dlg4* mRNA may act as a cofactor in such a UPS-related factor, and its (RNA) degradation may cause dysfunction of the factor, thereby leading to poor UPS control. It is of interest to identify protein(s) that bind to *Dlg4* mRNA and work as a UPS-related factor. FXR1 may be a candidate because it is associated with *Dlg4* mRNA and binds to proteasome (Figs. 3, 4). Further studies exploring candidate proteins, including FXR1, are needed to elucidate *Dlg4* mRNA-protein complex that works as a novel UPS-related factor.

*Dlg4* mRNA is also involved in neurite outgrowth (Figs. 5, 6). Interestingly, *Dlg4* mRNA appears to be expressed but translationally suppressed during early postnatal brain development, when neurite outgrowth is active (Fig. 6b, c). Similarly, in N2a cells, which can form neurite-like processes, *Dlg4* mRNA is expressed but the protein is hardly detected (Supplementary Fig. 2). Since gene silencing for *Dlg4* significantly suppresses neurite outgrowth and process formation (Figs. 5, 6), *Dlg4* mRNA may act as a functional RNA in neuritogenesis. Given that *Dlg4* mRNA is translationally suppressed during neurite outgrowth and then translated, it is conceivable that: *Dlg4* mRNA itself contributes to neurite outgrowth and the DLG4 protein, released from translational suppression, participates in synapse formation after neurite formation. Thus, it can be said that the *Dlg4* gene carries multiple genetic information and functions, as it is both a functional RNA and a template (information carrier) for protein synthesis.

Like *Dlg4*-knockdown, *Fxr1*-knockdown also showed a strong inhibition to neurite outgrowth and process formation (Figs. 5, 6), and both *Fxr1*- and *Dlg4*-knockdowns affected the UPS (Fig. 2). In neurons, the UPS plays important roles^24^. Thus, *Dlg4* mRNA may be involved in neuritogenesis through the UPS. Further studies are needed to determine whether *Dlg4* mRNA and FXR1 are involved in neuritogenesis as UPS-related factors.

Unlike *Fmr1-*, *Fxr1-* and *Dlg4*-knockdown, *Prnp*- and *Atf4*-knockdown has little impact on the UPS (Supplementary Fig. 4). This indicates that there is specificity in gene knockdown that affects the UPS, *i.e.*, some genes are associated with the UPS and some ones are not. Each such associated gene may contribute to regulation of the UPS by a different mechanism of action. For example, *Dlg4*-, *Fxr1*- and *Fmr1*-knockdown appears to result in different levels of ubiquitination (Fig. 2a). *Fxr1*- and *Fmr1*-knockdown increases proteasome activity (Fig. 2b), but FXR1 binds to proteasomes and FMRP does not (Fig. 4). These may provide evidence for different mechanisms of action. Different target proteins may be ubiquitinated by different gene knockdowns, and different E3 ubiquitin ligases may be involved in the ubiquitination; that is, FMRP, FXR1 and *Dlg4* mRNA may be associated with different E3 ubiquitin ligases. Furthermore, these UPS-related factors may affect the specificity and reactivity of proteolysis not only in proteasome but also in autophagy. These are of importance for understanding their mechanisms of action and need to be verified in the future.

Finally, this study has revealed a new role for FMRP, FXR1 and *Dlg4* mRNA as a UPS-related factor. These molecules are known to be involved in diseases and vital functions, but their association with the UPS was unknown. Therefore, we expect that this discovery will provide new clues to the investigation of the underlying causes of diseases in which these genes are involved and to the elucidation of their biological functions.

## Methods

### Cell culture

Neuro2a (N2a) cells, a mouse neuroblastoma cell line, were grown in alpha-minimum essential medium (α-MEM) (FUJIFILM Wako Pure Chemical Corp., Osaka, Japan) supplemented with 10% fetal bovine serum (Thermo Fisher Scientific, Waltham, MA, USA), 100 units/ml penicillin, and 100 μg/ml streptomycin (FUJIFILM Wako) at 37 °C in a 5% CO_2_ humidified chamber. For differentiation, N2a cells were trypsinized, diluted in the fresh medium and seeded onto BioCoat™ poly-D-lysine 6-well plates (CORNING Incorporated, Corning, NY, USA). After one-day incubation, the medium was changed to serum-free α-MEM with or without 2 × 10^–7^ M all-*trans*-retinoic acid (RA) (Sigma-Aldrich Japan, Tokyo, Japan), and the cells were further incubated at 37 °C. For chemical treatment, cells were treated by adding 100 μM cycloheximide (FUJIFILM Wako), 1 μM MG132 (FUJIFILM Wako), and 200 ng/ml rapamycin (Bio-techne, Minneapolis, MN, USA) in the medium.

Primary culture of mouse hippocampal neurons was performed as described previously with minor modifications. Briefly, mouse E16 embryonic hippocampal tissue (C57BL/6 J mouse strain) was isolated, treated with 0.25% trypsin–EDTA (FUJIFILM Wako) at 37 °C for 10 min and dissociated by gentle pipetting. Dissociated hippocampal neurons were seeded onto BioCoat™ poly-D-lysine 6-well plates (CORNING) and incubated in D-MEM (High Glucose) with L-Glutamine, Phenol Red and Sodium Pyruvate (FUJIFILM Wako) supplemented with 10% fetal bovine serum (Thermo Fisher Scientific) at 37 °C in a 5% CO_2_ humidified chamber. After one-day incubation, the medium was changed to Neurobasal™ medium (Thermo Fisher Scientific) supplemented with B27 supplement (Thermo Fisher Scientific) and 0.5 mM L-Alanyl-L-Glutamine (FUJIFILM Wako). Neurons were grown at 37 °C in a 5% CO_2_ humidified chamber.

### RNA and DNA oligonucleotides

RNA and DNA oligonucleotides used in this study were synthesized by and purchased from Hokkaido System Science (Sapporo, Hokkaido, Japan) or Sigma-Aldrich. The sequences of synthesized oligonucleotides are shown in Supplementary Tables 1 and 2. Stealth siRNAs for Fxr1 (MSS204455, MSS204457) were purchased from Thermo Fisher Scientific.

### Plasmids

For gene editing, the pX330-U6-Chimeric_BB-CBh-hSpCas9 plasmid (Addgene, Cambridge, CA, USA) was digested by *Bbs* I (New England BioLabs, Ipswich, MA, USA) and designed oligoDNA duplex encoding guide RNA sequence against the *Fmr1* gene was inserted to the digestion site of the plasmid. The sequences of designed DNA oligonucleotides are indicated in Supplementary Table 1. The pd2EGFP-N1 plasmid encoding the *green fluorescent protein* (*GFP*) gene was purchased from Clontech Laboratories (Palo Alto, CA, USA) and used as a reporter.

### Total RNA and genomic DNA extraction

Total RNA and genomic DNA were extracted from cells using a TRI Reagent [Molecular Research Center (MRC), Cincinnati, OH, USA] and a Wizard SV genomic DNA purification system (Promega, Madison, WI, USA), respectively, according to the manufacturers’ instructions.

### Transfection

The day before transfection, N2a cells were trypsinized and seeded onto BioCoat™ poly-D-lysine 6-well plates (CORNING). The pd2EGFP-N1 plasmid (1.25 µg/well) and/or synthetic siRNAs (100 pmol/well) were transfected into N2a cells and primary hippocampal neurons using a Lipofectamine2000 and a Lipofectamine3000 transfection reagents (Thermo Fisher Scientific), respectively, according to the manufacturer's instructions. 24 h after transfection, medium was changed to fresh medium, and the cells were further incubated. Cells, in which GFP was expressed from the pd2EGFP-N1 plasmid, were examined by a fluorescent microscope [Axiovert 40 CFL (Carl Zeiss, Jena, Germany). The sequences of the siRNAs used are shown in Supplementary Table 2.

### Establishment of FMRP-deficient N2a cell

N2a cells were seeded onto 100 mm tissue-culture dishes (~ 2 × 10^3^ cells/dish) and subjected to transfection of the constructed pX330 plasmid (see above) (10 µg) using a Lipofectamine2000 transfection reagent (Thermo Fisher Scientific) according to the manufacturer's instructions. Five days after transfection, each cell colony was collected, trypsinized, diluted in fresh medium and reseeded onto 96-well culture plates. After cell growth, genomic DNA was prepared from a portion of the cells in each well and the *Fmr1* gene was examined by PCR*.* To obtain cloned FMRP-deficient cells, dilution of candidate cells, reseeding and examination by PCR were repeated. The sequences of PCR primers are indicated in Supplementary Table 1.

### Protein synthesis assay and translational inhibition

Overall nascent proteins were examined by a EZClick™ global protein synthesis assay kit (BioVision, Waltham, MA, USA) according to the manufacturer’s instructions. N2a cells were seeded onto 96-well culture plates (1 × 10^4^ cells/well) coated with poly-l-lysine (Nacalai tesque, Kyoto, Japan). The cells were transfected with siDLG4, siFXR1, siFMR1 and siCont (1 pmol/well) and incubated at 37 °C for 24 h. The culture medium was replaced with fresh medium containing EZClick™ protein label, and cells were further incubated at 37 °C for 24 h. After incubation, the cells were fixed, permeabilized, and treated with the reaction cocktail for 30 min in the dark according to the manufacturer’s instructions. The treated cells were washed five times in the wash buffer of the kit and examined in bottom measurement mode using a Synergy H1 Multi-Mode Reader (BioTeK, Winooski, VT, USA). For data correction, the total DNA content of each well was measured according to the manufacturer’s instructions.

Cycloheximide (FUJIFILM Wako) was used as a translation inhibitor. Cells were treated with 100 µM cycloheximide for 9 h at 37 °C in a 5% CO_2_ humidified chamber.

### Ubiquitination and proteasome assay

Ubiquitination and proteasome activities were examined using a CycLex poly-ubiquitinated protein ELISA kit [Medical & Biological Laboratories (MBL), Tokyo, Japan] and a CycLex proteasome enrichment & activity assay kit (MBL), respectively, according to the manufacturer’s instructions. Briefly, cells were lysed with the provided cell extraction buffer and the lysate was centrifuged at 15,000 rpm at 4 °C according to the manufacturer’s instructions. For data correction, aliquots were taken from the supernatant and subjected to protein assay using a Protein quantification kit (DOJINDO, Kumamoto, Japan) according to the manufacturer’s instructions. Supernatants containing equal amounts of protein were used for the ubiquitination and proteasome assays according to the manufacturer’s instructions. The levels of ubiquitination and proteasome activities were measured by a Synergy H1 Multi-Mode Reader (BioTeK). To see the proteins bound to proteasomes, the proteasome samples collected were further examined by western blotting.

### Quantification of p62

The p62 protein was quantified using a CycLex Total p62 ELISA Kit (MBL) according to the manufacturer’s instructions. Briefly, cells were lysed with the provided lysis buffer, and the lysate was centrifuged at 13,000 rpm for 5 min at 4 °C. For data correction, aliquots were taken from the supernatant and subjected to protein assay as described above. The rest supernatants containing equal amounts of protein were used for the p62 ELISA according to the manufacturer’s instructions. The levels of p62 were measured by a Synergy H1 Multi-Mode Reader (BioTeK).

### Immunoprecipitation

Immunoprecipitation was performed as described previously (Fukuoka et al. 2018). Briefly, FMRP-deficient N2a cells were homogenized in 1 mL of ice-cold lysis buffer [10 mM HEPES (pH7.4), 200 mM NaCl, 30 mM EDTA, 0.5% Triton-X100, 0.4U/μl RNasin Plus RNase inhibitor (Promega), 1 × Protease/Phosphatase inhibitor Cocktail (Cell Signaling Technology, Danvers, MA, USA)]. Lysate was centrifuged at 3000 × g for 10 min at 4 °C, and the supernatant was transferred into a new tube. The NaCl concentration of the supernatant was adjusted to 400 mM. A portion of the supernatant was taken and stored as a crude lysate sample. The rest was centrifuged at 70,000 × g for 20 min at 4 °C, and the supernatant was mixed with yeast tRNAs (Sigma-Aldrich) and pretreated with protein A-Sepharose 4B slurry (Sigma-Aldrich) for 30 min at 4 °C. After centrifugation at 12,000 × g for 2 min at 4 °C, the supernatant was divided equally. The divided samples were added with anti-FXR1 antibody (Cat no: 13194–1-AP; Proteintech, Rosemont, IL, USA) and a control IgG (Cat no: 3900S; Cell Signaling Technology), respectively, and gently mixed using a rotator overnight at 4 °C. Protein A-Sepharose 4B slurry was added to the treated samples and further incubated at 4 °C for 1 h with gentle mixing. After incubation, the protein A-Sepharose beads were collected by centrifugation at 12,000 × g for 2 min at 4 °C, washed four times with the lysis buffer containing yeast tRNAs, and finally washed in the lysis buffer. RNAs co-immunoprecipitated with antibodies (protein A-Sepharose beads) were extracted using a TRI Reagent (MRC) according to the manufacturer’s instructions.

### Quantitative reverse transcription-polymerase chain reaction (qRT-PCR)

Total RNA was subjected to complementary DNA (cDNA) synthesis using oligo (dT)_15_ primers (Promega) and a SuperScript® III reverse transcriptase (Thermo Fisher Scientific) according to the manufacturer's instructions. Resulting cDNAs were subjected to quantitative PCR analysis using a StepOne Plus Real-Time PCR system (Thermo Fisher Scientific) with a FastStart Universal SYBR Green Master (Rox) (Roche, Basel, Switzerland) and Perfect Real Time primers (TAKARA BIO, Shiga, Japan) or synthesized PCR primers according to the manufacturer's instructions. The Perfect Real Time primers used are as follows (TAKARA BIO primer-set ID):

*Fmr1* (MA104840), *Fxr1* (MA140271), *Dlg4* (MA105913), *Atf4* (MA101866) and *Gapdh* (MA050371).

PCR primers for *EGFP* was chemically synthesized and the sequences of the primers are indicated in Supplementary Table 1.

### Western blot

N2a cells, primary neurons and brain tissues were lysed in lysis buffer [20 mM Tris–HCl (pH 7.5), 150 mM NaCl, 1 mM EGTA, 1% Triton X-100] containing 1 × protease inhibitor cocktail (Roche). The lysate was centrifuged at 1000 × g for 15 min at 4 °C. The supernatant was collected, and protein concentration was measured by a Protein quantification kit (DOJINDO) according to the manufacturer’s instructions. Equal amounts of protein were mixed with 1/4 volume of the sample buffer (0.25 M Tris–HCl, pH6.8, 40% glycerol, 8% SDS, 0.04% bromophenol blue, 16% beta-mercaptoethanol), and boiled at 100 °C for 5 min. For nucleoprotein, the precipitated pellets were dissolved in 4 × diluted sample buffer and subjected to three times pules sonication (10 s sonication at a 30% amplitude and 5 s resting interval) on ice using a QSONICA Q125 (WAKEN B TECH, Tokyo, Japan), followed by boiling as above. The protein samples were electrophoretically separated on 8% or 10% SDS–polyacrylamide gels and blotted onto polyvinylidene fluoride membranes (Immobilon P) (Millipore, Billerica, MA, USA). The membranes were subjected to blocking in blocking solution (5% BSA in TBS-T buffer [Tris-buffered saline (TBS) containing 0.1% Tween 20]) for 1 h and incubated with diluted primary antibodies at 4 °C overnight. After incubation, the membranes were washed in TBS-T buffer and incubated with 1/8000 diluted horseradish peroxidase (HRP)-conjugated goat anti-mouse IgG (cat no: 7076, Cell Signaling Technology), HRP-conjugated goat anti-rabbit IgG (cat no: 7074, Cell Signaling Technology) or HRP-conjugated rabbit anti-goat IgG (cat no: sc-2768, Santa Cruz Biotechnology, Texas, USA) for 1 h at room temperature. Antigen–antibody complexes were visualized using an ECL Prime Western Blotting Detection Reagent (Cytiva, Tokyo, Japan) according to the manufacturer's instructions and detected by an ImageQuant™ LAS 500 (GE Healthcare Bio-Sciences AB, Uppsala, Sweden) according to the manufacturer's instructions. The original membranes, detected signals and merged images (automatically merged by LAS 500) are shown in Supplementary Information as original blots. Signal intensities of bands were measured by the ImageJ software. The primary antibodies used are indicated below with the product IDs and dilution ratios in parentheses:

Anti-DLG4 (ab2723, 1/2000), anti-FMRP (ab17722, 1/2000) and anti-PRNP (ab52604, 1/5000) were purchased from Abcam (Cambridge, UK). Anti-FXR1 (13,194–1-AP, 1/5000), anti-PSMA7 (15,219–1-AP, 1/5000) and anti-βIII-TUBULIN (66,375–1-Ig, 1/5000) were purchased from Proteintech. Anti-GAPDH (2118S, 1/2000) and anti-ATF4 (11815S, 1/2000) were purchased from Cell Signaling Technology. Anti-LAMIN A (SC-6214, 1/2000) and anti-ACTIN (SC-1616, 1/2000) were purchased from Santa Cruz Biotechnology. Anti-GFP (A-11122, 1/2000) and Anti-α-TUBULIN (F2168, 1/2000) were purchased from Thermo Fisher Scientific and Sigma-Aldrich, respectively.

### Statistics and reproducibility

Data obtained in this study were initially evaluated by one-way analysis of variance (ANOVA) or Welch's *t* test. If significant difference between data was detected by ANOVA, Tukey’s post hoc test or Dunnett’s test was carried out between the data of interest. The level of statistical significance was set at 0.05.

## Supplementary Information

Supplementary Information 1.

Supplementary Information 2.

Supplementary Information 3.

Supplementary Information 4.

## Data Availability

All data generated or analyzed during this study are included in this published article (and its Supplementary information files), and the source data underlying the graphs are provided in Supplementary Data.
