# Supplementary material for: FMRP, FXR1 protein and Dlg4 mRNA, which are associated with fragile X syndrome, are involved in the ubiquitin–proteasome system
Source: Sci Rep. 2023 Feb 2;13:1956. doi: 10.1038/s41598-023-29152-4 (PMC9894842; doi:10.1038/s41598-023-29152-4)
Supplement: Supplementary file 1 — Supplementary Information 1. [file 41598_2023_29152_MOESM1_ESM.pdf]

FMRP, FXR1 protein and *Dlg4* mRNA, which are associated with Fragile X syndrome, are involved in the ubiquitin-proteasome system

Hideo Shimizu[1] and Hirohiko Hohjoh[1,*]

[1]Department of Molecular Pharmacology, National Institute of Neuroscience, NCNP, Tokyo, Japan

***Corresponding Author**: Hirohiko Hohjoh, Ph.D.
Department of Molecular Pharmacology, National Institute of Neuroscience, NCNP 4-1-1 Ogawahigashi, Kodaira, Tokyo 187-8502, JAPAN
Tel: +81-42-342-2711, ext. 5234, Fax: +81-42-346-3594
E-mail: hohjohh@ncnp.go.jp

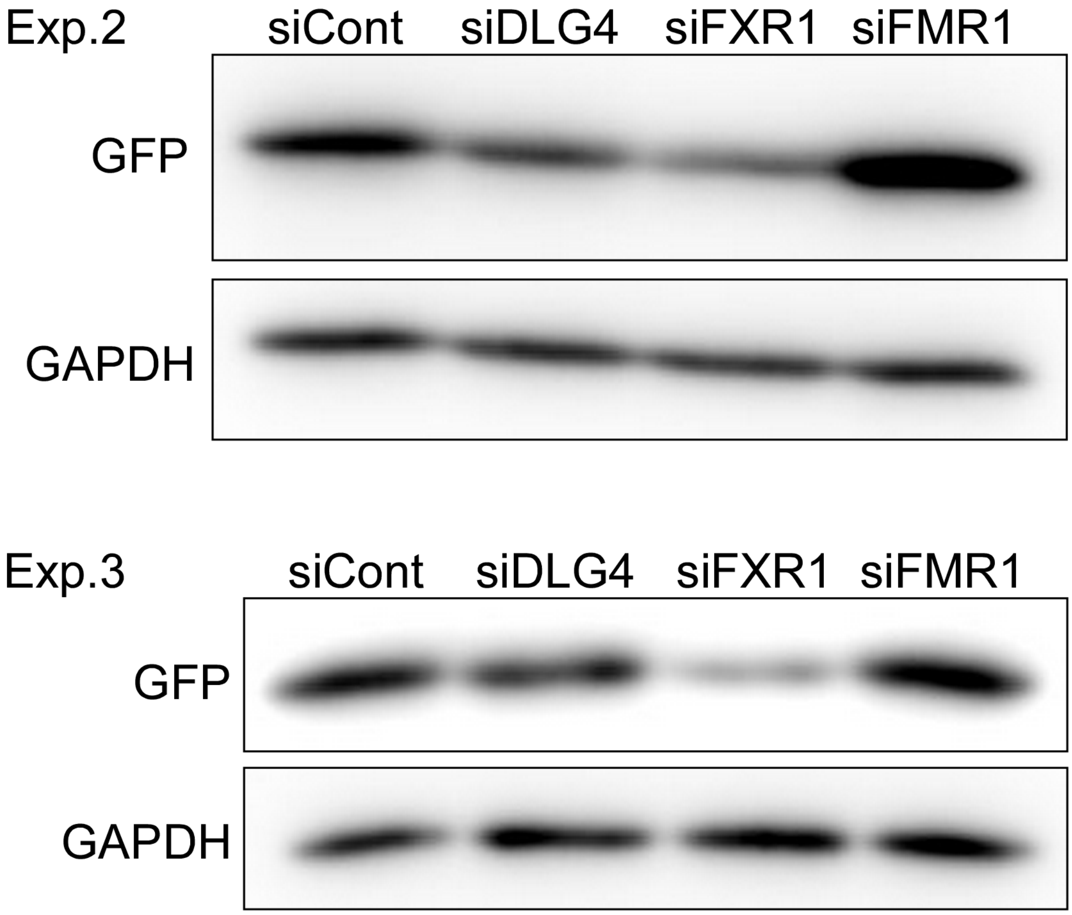

Supplementary Fig. 1. GFP expression under *Dlg4* -, *Fxr1* - and *Fmr1* -knockdown conditions. Repeating the experiment in Fig. 1a (Experiment 1), the results of Experiments 2 and 3 (Exp. 2, 3) are presented above.

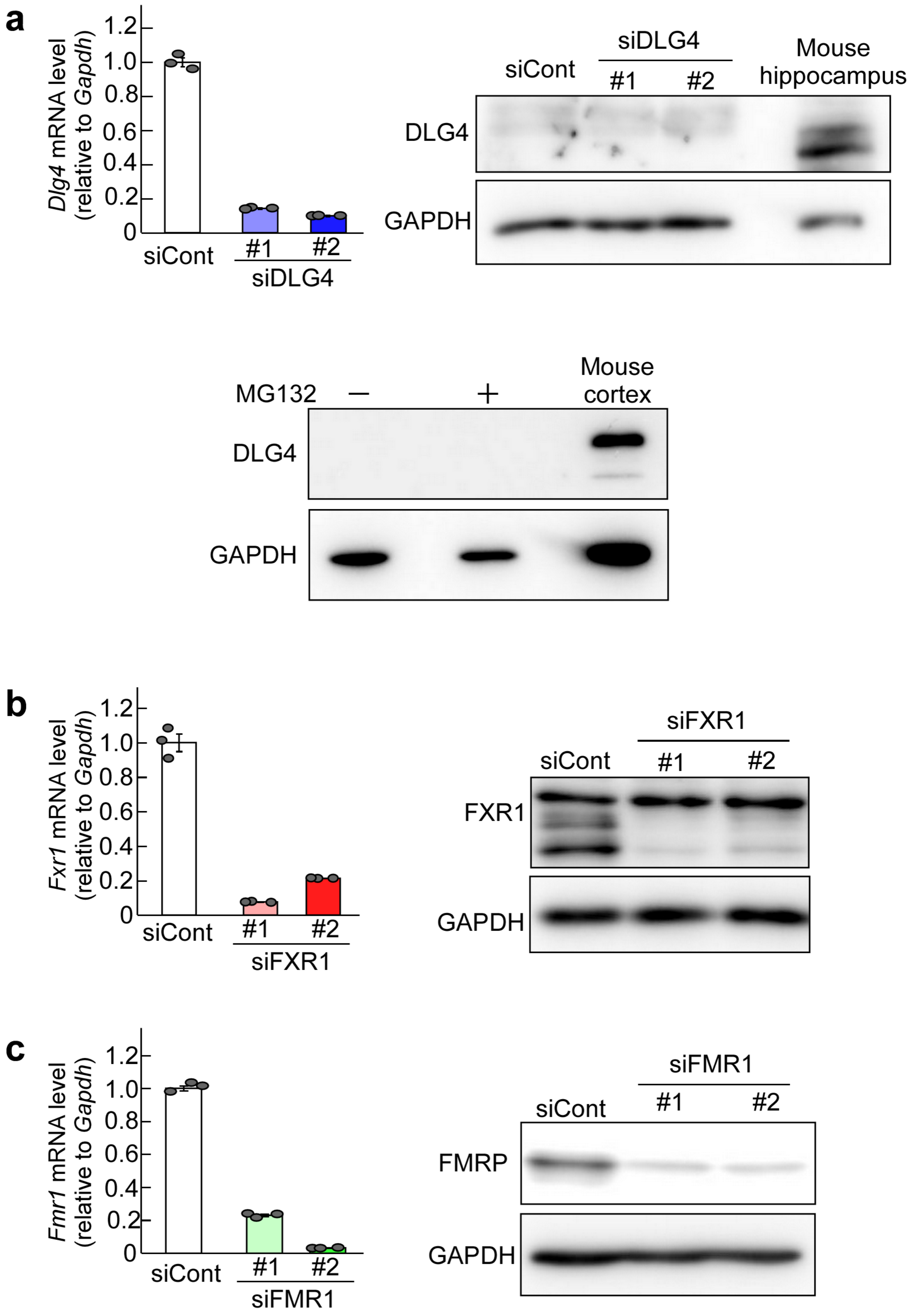
a
Dlg4 mRNA level (relative to Gapdh)
1.2
1.0
0.8
0.6
0.4
0.2
0
siCont
#1
#2
siDLG4
siCont
siDLG4
#1
#2
Mouse hippocampus
DLG4
GAPDH
MG132
−
+
Mouse cortex
DLG4
GAPDH
b
Fxr1 mRNA level (relative to Gapdh)
1.2
1.0
0.8
0.6
0.4
0.2
0
siCont
#1
#2
siFXR1
siFXR1
siCont
#1
#2
FXR1
GAPDH
c
Fmr1 mRNA level (relative to Gapdh)
1.2
1.0
0.8
0.6
0.4
0.2
0
siCont
#1
#2
siFMR1
siFMR1
siCont
#1
#2
FMRP
GAPDH

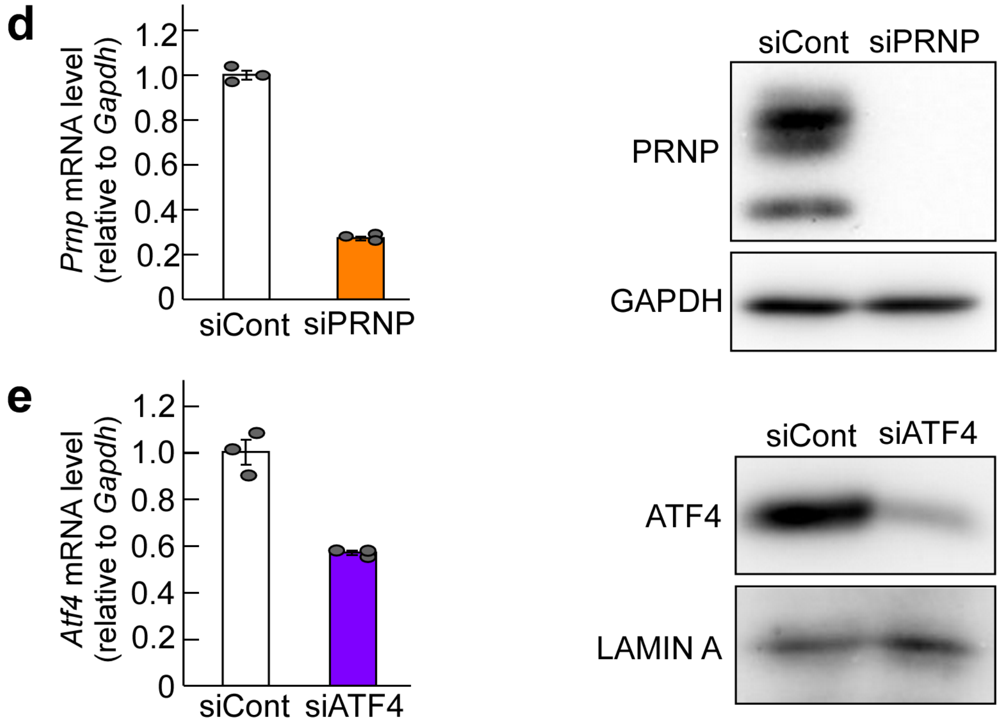

Supplementary Fig. 2. Gene expression and gene knockdown (silencing) in N2a cells. N2a cells were subjected to gene knockdown with siRNAs used in this study (see Supplementary Table 2) and the target gene transcripts (mRNAs) and the proteins were examined by qRT-PCR and western blotting, respectively. The *Gapdh* gene was examined as an internal control. The qRT-PCR data were analyzed by the delta-delta Ct method using the data of *Gapdh* as a reference, and further normalized with the data obtained from cells treated with non-silencing control siRNA (siCont) as 1. Data are shown as mean ± SEM (n = 3 independent determination). **a** *Dlg4* expression and gene silencing. The siRNAs used are indicated. Mouse hippocampus (tissue extract) was examined as a positive control in western blotting (right panel). *Dlg4* mRNA is present and is significantly reduced by gene silencing, but the protein is hardly detected even in siCont-treated N2a cells. Furthermore, DLG4 protein could not be detected in N2a cells treated with MG132, a proteasome inhibitor. These results suggest that *Dlg4* mRNA is translationally suppressed. **b** *Fxr1* expression and gene silencing. **c** *Fmr1* expression and gene silencing. **d** *Prnp* expression and gene silencing. **e** *Atf4* expression and gene silencing. ATF4 protein is a nuclear protein. LAMIN A, also a nuclear protein, was examined as a loading control in western blotting.

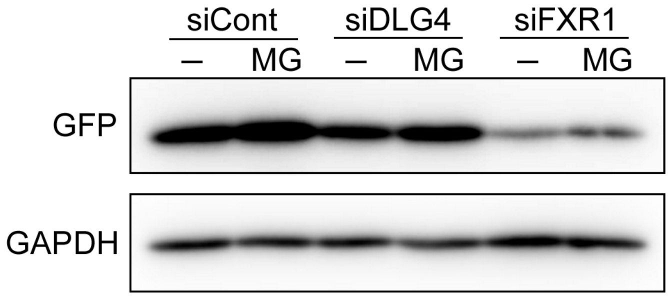

Supplementary Fig. 3. MG132 treatment. N2a cells were transfected with the *GFP* gene as a reporter together with the indicated siRNAs and incubated for 42 h. The cells were treated with MG132 (MG), a proteasome inhibitor, and its vehicle (DMSO) (-) for 6 h, and cell lysate was prepared. GFP and GAPDH as a loading control were examined by western blotting as in Fig. 1. The lower intensity of GFP in the *Fxr1*-knockdown condition compared to the *Dlg4*-knockdown and control conditions, even in MG132 treatment, may be due to selective autophagy activated under *Fxr1*-knockdown conditions, which may be involved in the proteolysis of GFP (see Fig. 2d).

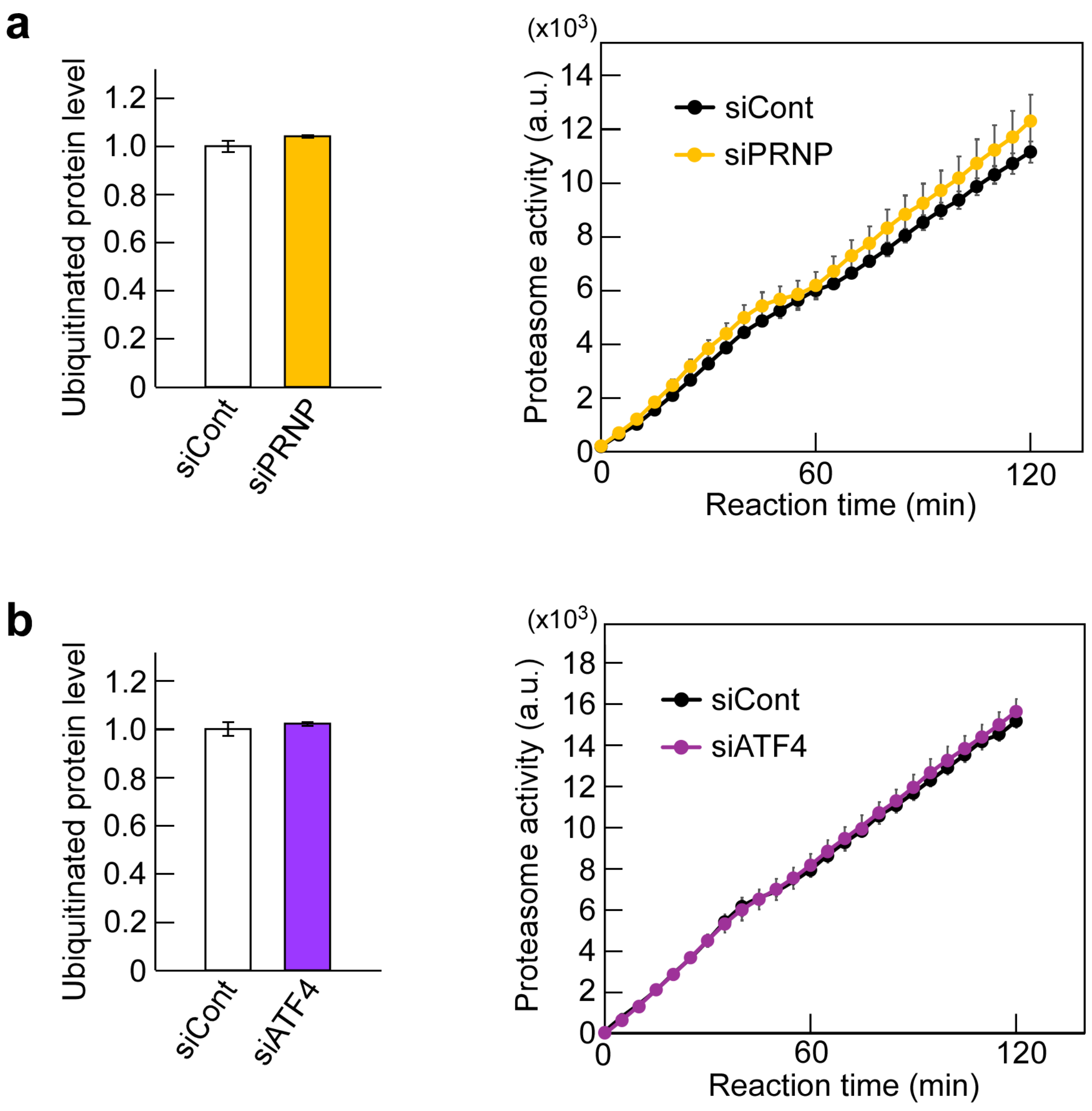

Supplementary Fig. 4. Ubiquitination and proteasome activity under *Prnp*- and *Atf4*-knockdown conditions. N2a cells were subjected to gene knockdown with the indicated siRNAs, and ubiquitination and proteasome activity were examined and analyzed as in Fig. 2. Data are shown as mean ± SEM (n = 3 independent determination).

Supplementary figure 5

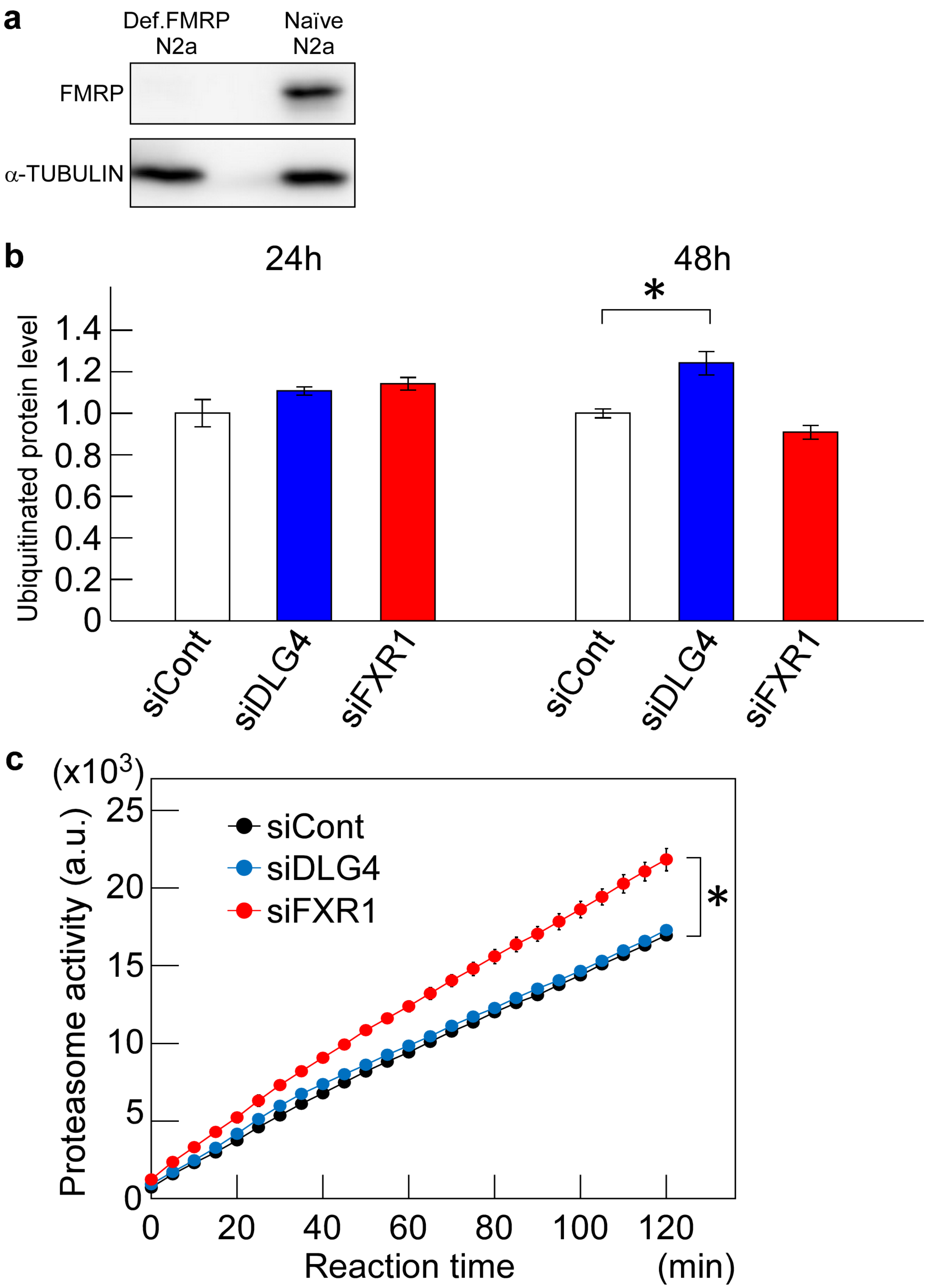

Supplementary Fig. 5. Property of FMRP-deficient (def.FMRP) N2a cell. **a** Western blotting for FMRP. Naïve N2a cells and def.FMRP-N2a cells were examined by western blotting for FMRP as in Supplementary Fig. 2c. **b, c** Ubiquitination and proteasome activity in def.FMRP-N2a cells. Def.FMRP-N2a cells were subjected to gene knockdown with the indicated siRNAs, and ubiquitination (**b**) and proteasome activity (**c**) were examined and analyzed as in Fig. 2. Data are shown as mean ± SEM (n = 3 independent determination). The levels of proteasome activity are indicated at arbitrary units (a.u.). The data were compared to the data obtained from siCont-treated cells (**b**, $^*P < 0.05$ by one-way analysis of variance with Dunnett's t test; **c**, $^*P < 0.05$ by two-way analysis of variance).

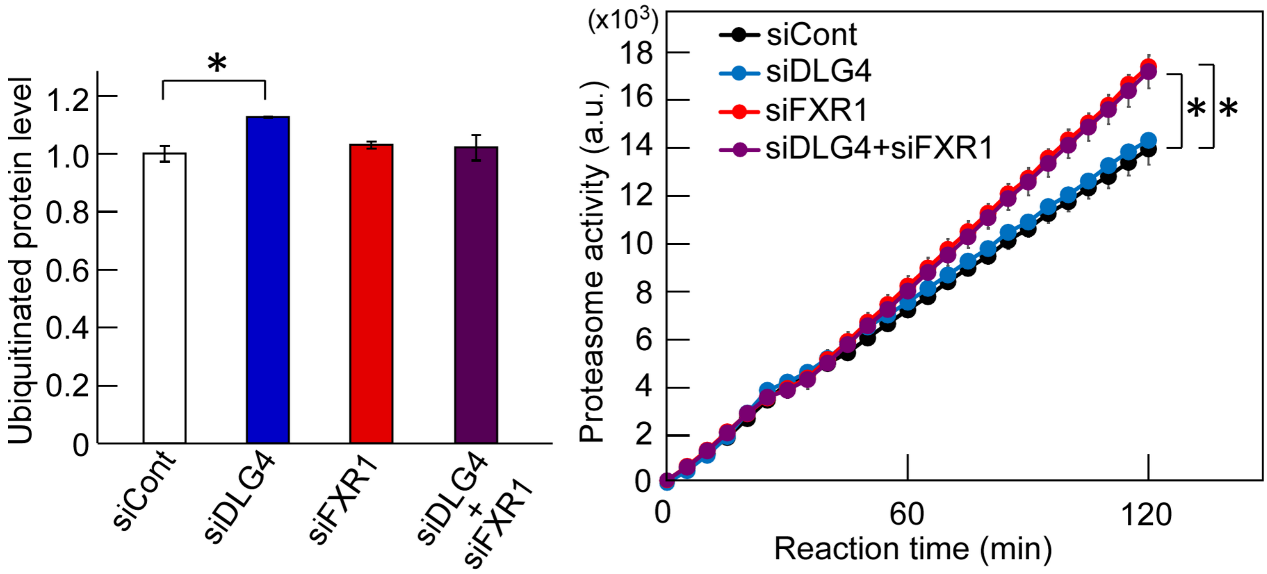

Supplementary Fig. 6. Ubiquitination and proteasome activity under *Dlg4* and *Fxr1* double-knockdown conditions. N2a cells were subjected to single-gene knockdown with siDLG4 or siFXR1 and double-gene knockdown with siDLG4 and siFXR1. Ubiquitination and proteasome activity were examined and analyzed as in Fig. 2. Data are shown as mean ± SEM (n = 3 independent determination). The levels of proteasome activity are indicated at arbitrary units (a.u.). The data were compared to the data obtained from siCont-treated cells (ubiquitination, *P < 0.05 by one-way analysis of variance with Dunnett's t test; proteasome activity, *P < 0.05 by two-way analysis of variance).

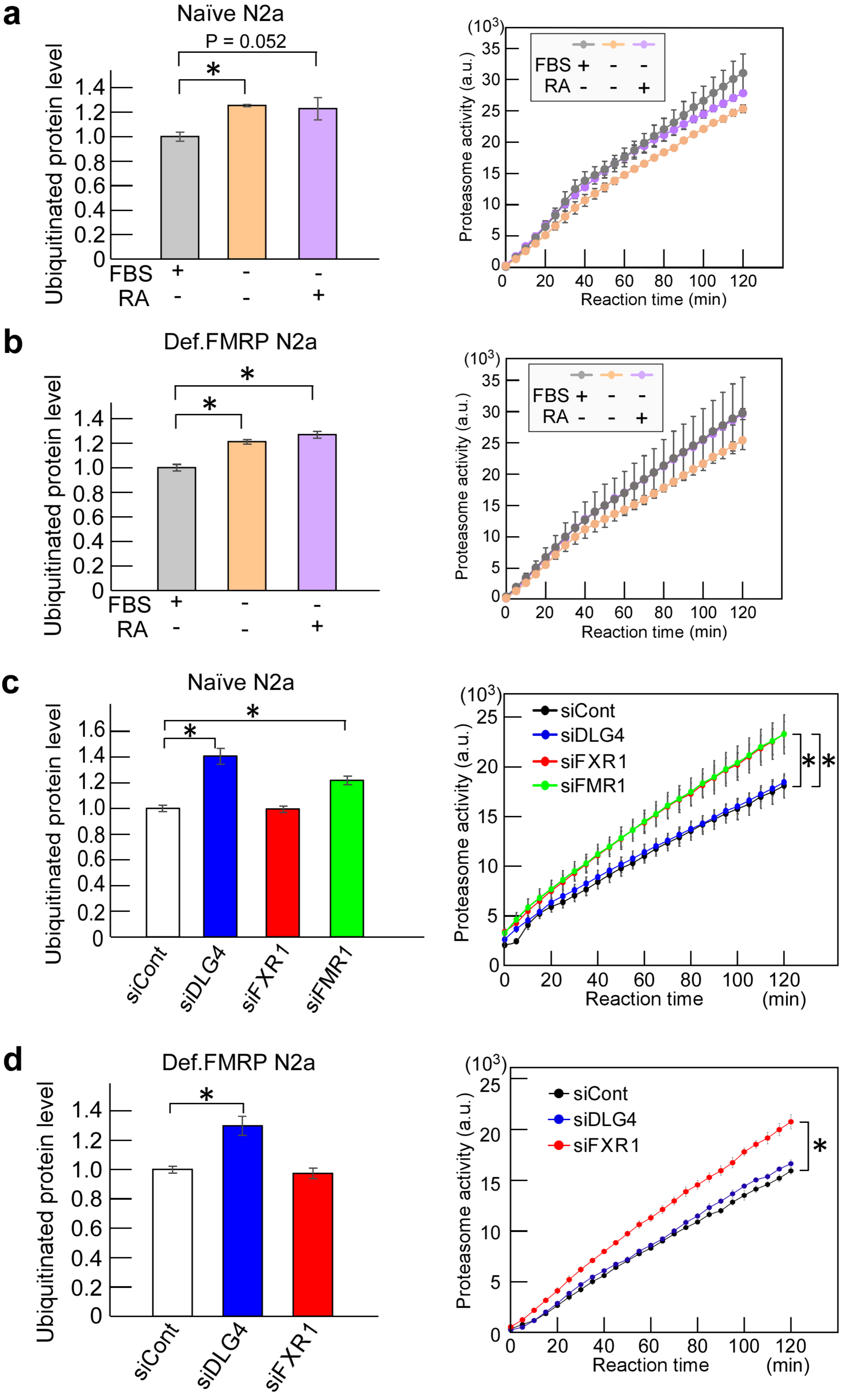
a
Naïve N2a
P = 0.052
*
Ubiquitinated protein level
1.4
1.2
1.0
0.8
0.6
0.4
0.2
0
FBS + - -
RA - - +
(10^3)
35
30
25
20
15
10
5
0
FBS + - -
RA - - +
Proteasome activity (a.u.)
0 20 40 60 80 100 120
Reaction time (min)
b
Def.FMRP N2a
*
*
Ubiquitinated protein level
1.4
1.2
1.0
0.8
0.6
0.4
0.2
0
FBS + - -
RA - - +
(10^3)
35
30
25
20
15
10
5
0
FBS + - -
RA - - +
Proteasome activity (a.u.)
0 20 40 60 80 100 120
Reaction time (min)
c
Naïve N2a
*
*
Ubiquitinated protein level
1.6
1.4
1.2
1.0
0.8
0.6
0.4
0.2
0
siCont
siDLG4
siFXR1
siFMR1
(10^3)
25
20
15
10
5
0
siCont
siDLG4
siFXR1
siFMR1
*
*
Proteasome activity (a.u.)
0 20 40 60 80 100 120
Reaction time
(min)
d
Def.FMRP N2a
*
Ubiquitinated protein level
1.4
1.2
1.0
0.8
0.6
0.4
0.2
0
siCont
siDLG4
siFXR1
(10^3)
25
20
15
10
5
0
siCont
siDLG4
siFXR1
*
Proteasome activity (a.u.)
0 20 40 60 80 100 120
Reaction time
(min)

Supplementary Fig. 7. UPS activity under differentiation conditions. Naïve N2a (**a**) and def.FMRP-N2a (**b**) cells were cultured under serum-free (FBS-) and retinoic acid (RA+)-added culture conditions (differentiation conditions) and normal culture conditions (FBS+) for 24 h, and intracellular ubiquitination and proteasome activity were examined as in Fig. 2. In addition, naïve N2a (**c**) and def.FMRP-N2a (**d**) cells were subjected to gene knockdown with the indicated siRNAs, cultured for 48 h under differentiation conditions, and examined for intracellular ubiquitination and proteasome activity as in Fig. 2. Data are shown as mean ± SEM (n = 3 independent determination). The levels of proteasome activity are indicated at arbitrary units (a.u.). The data were compared to the data obtained from siCont-treated cells (ubiquitination, $*P < 0.05$ by one-way analysis of variance with Dunnett's t test; proteasome activity, $*P < 0.05$ by two-way analysis of variance).

Original blots.

The original blot images in Figures are shown on the following pages. In the Figure, the blot images indicated by red boxes have been used. The leftmost pictures show the membranes used for western blotting, and the signals detected from the membranes and merged images (automatically merged by LAS 500, a CCD camera system) are shown. The membranes were never cut prior to reaction with antibodies. Arrows indicate the process of stripping the antibody and reacting the next antibody.

Original blot 1

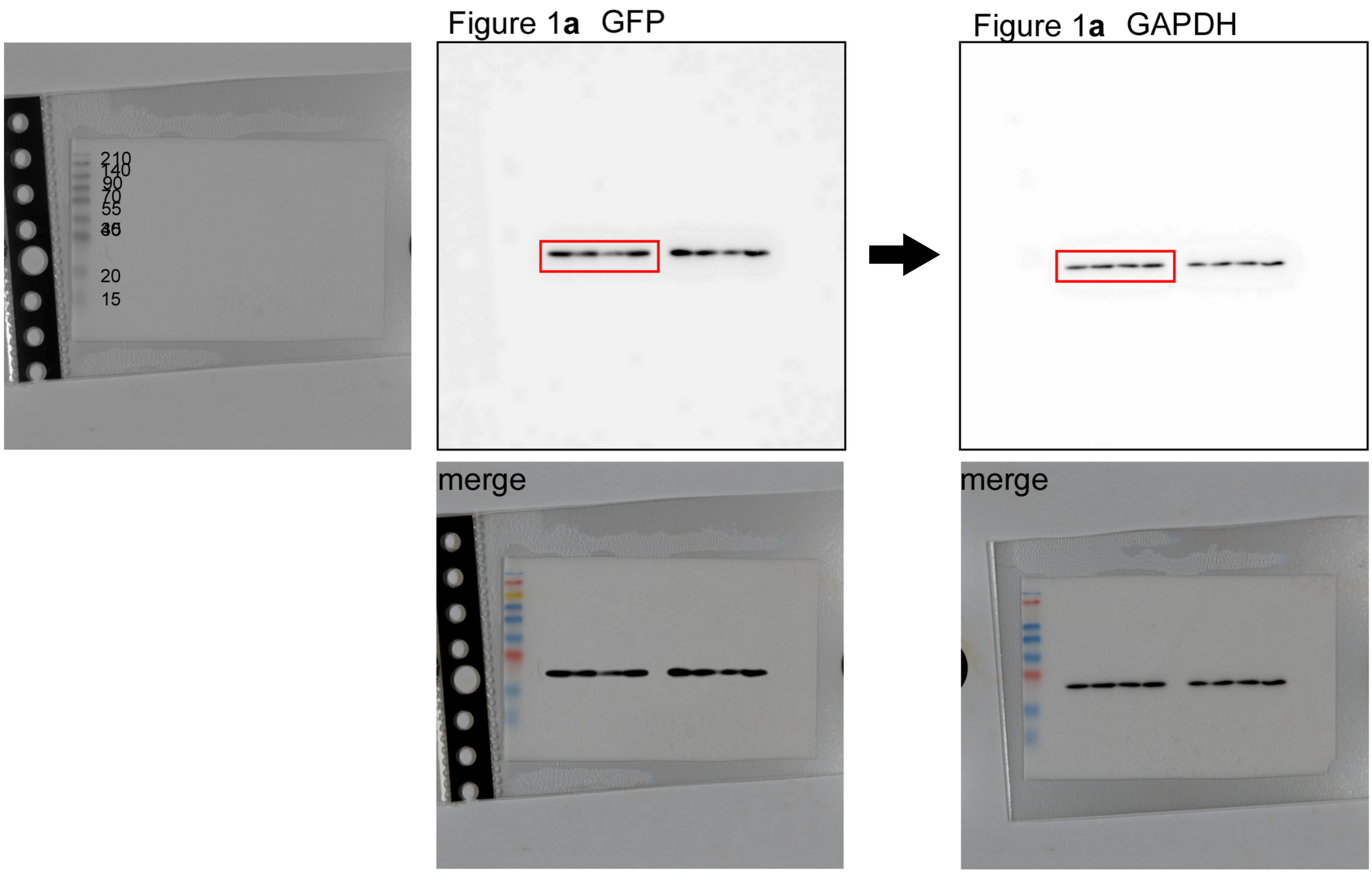

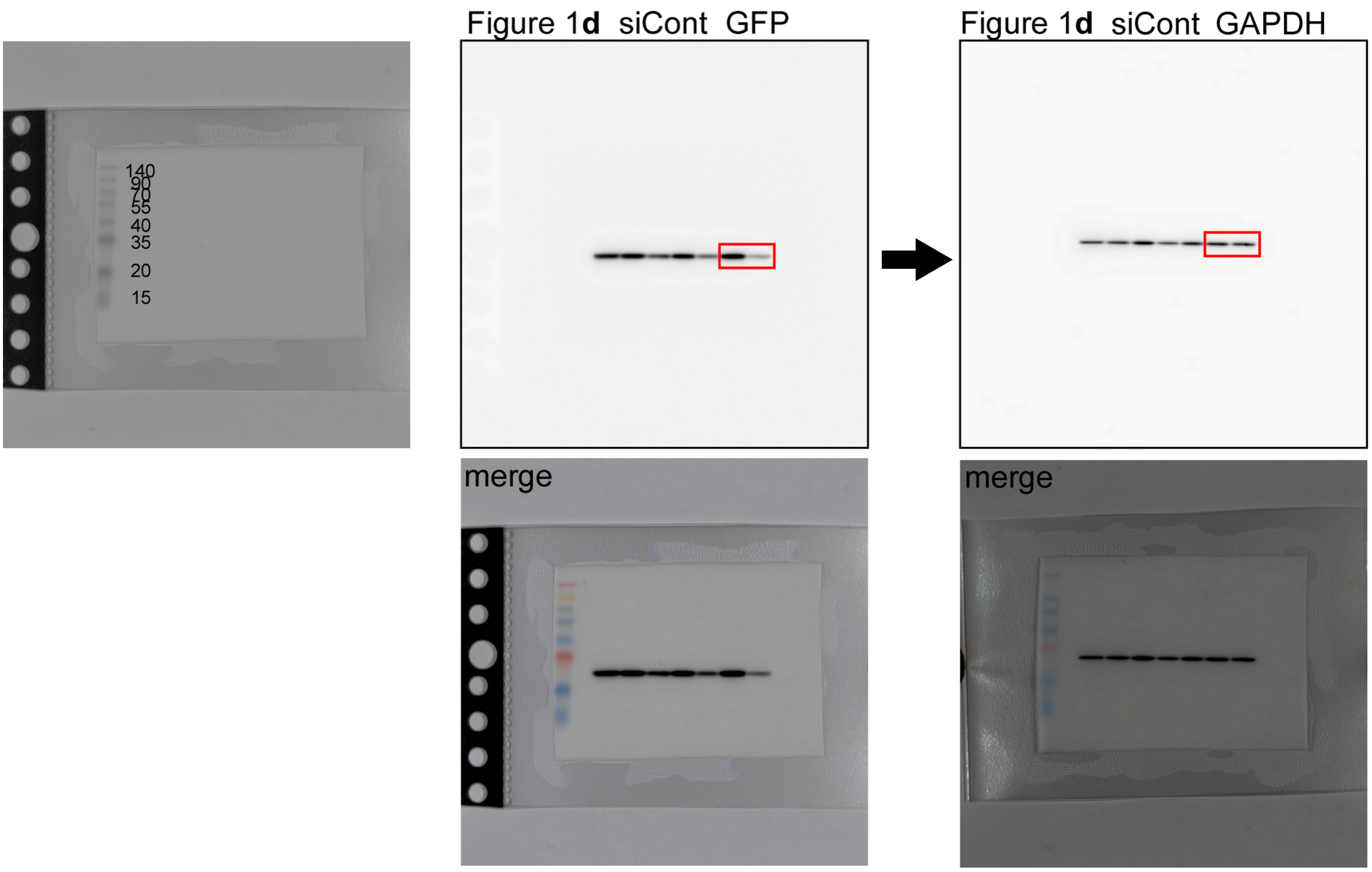

Original blot 2

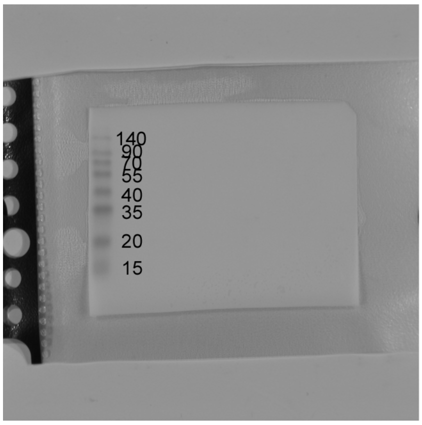

Figure 1**d** siDLG4 GFP

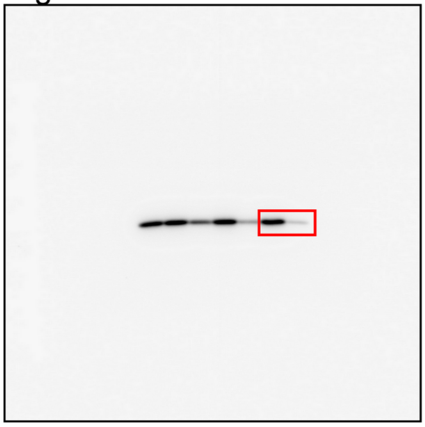

Figure 1**d** siDLG4 GAPDH

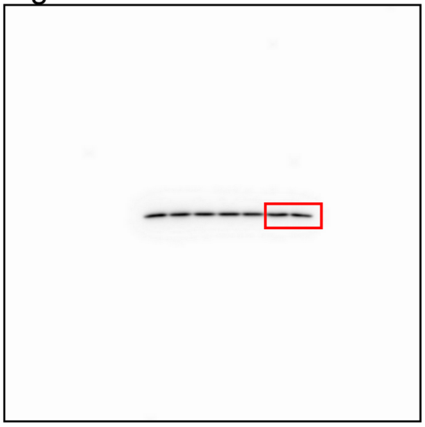

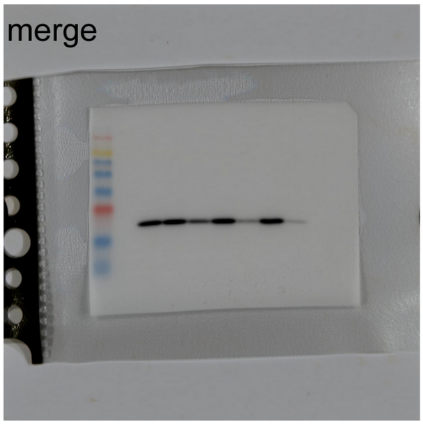

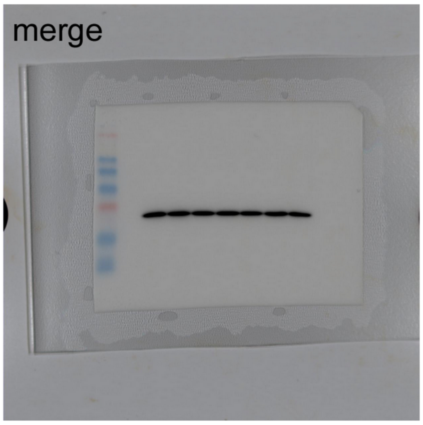

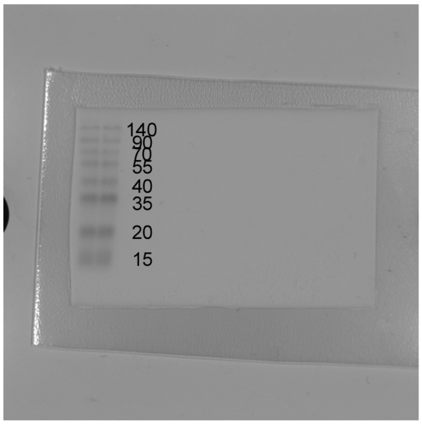

Figure 1**d** siFXR1 GFP

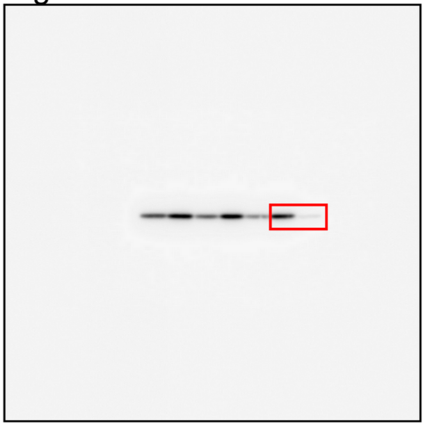

Figure 1**d** siFXR1 GAPDH

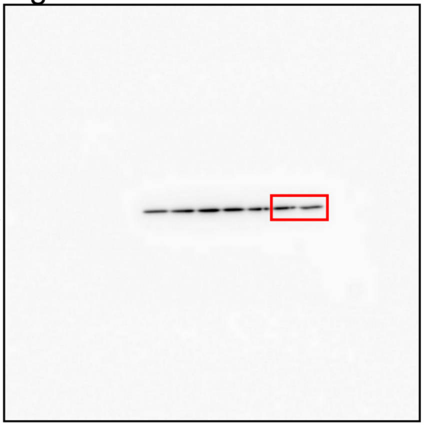

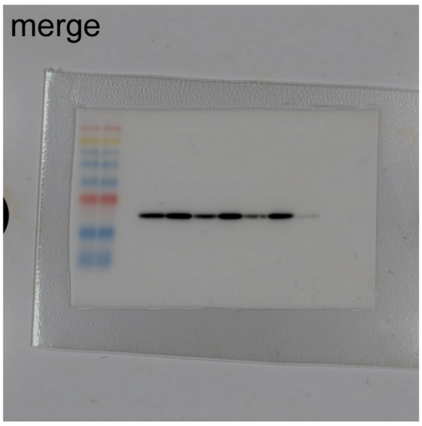

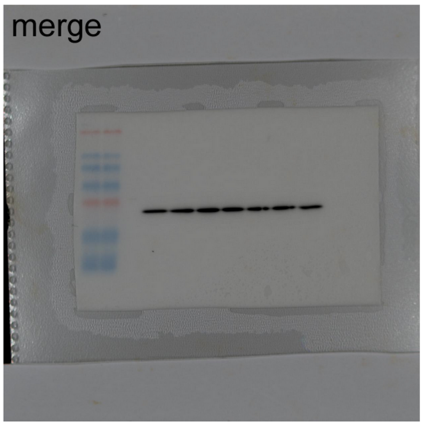

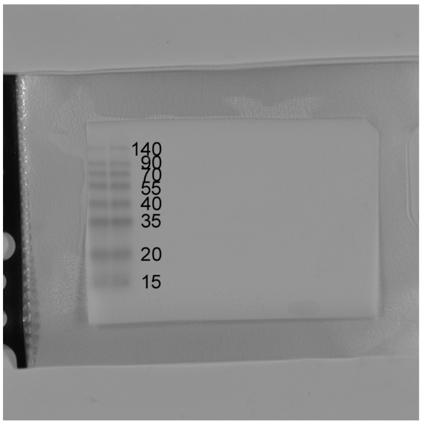

Figure 1**d** siFMR1 GFP

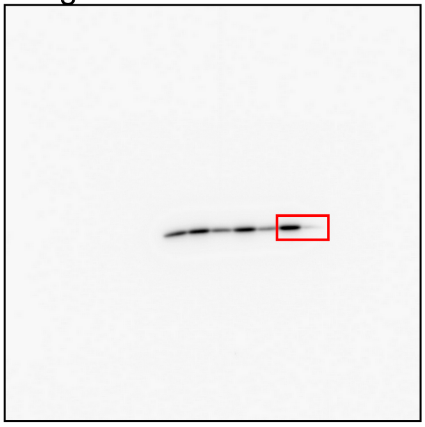

Figure 1**d** siFMR1 GAPDH

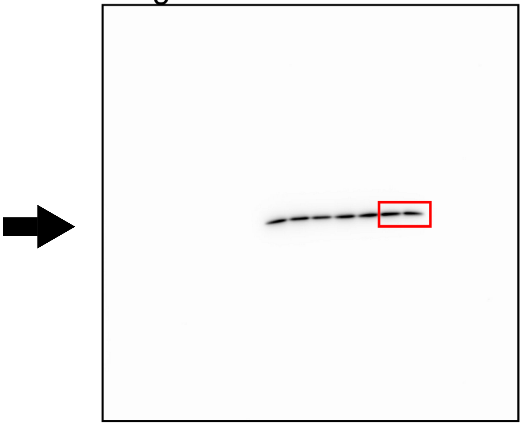

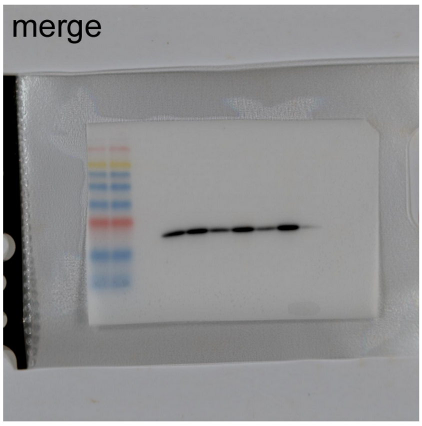

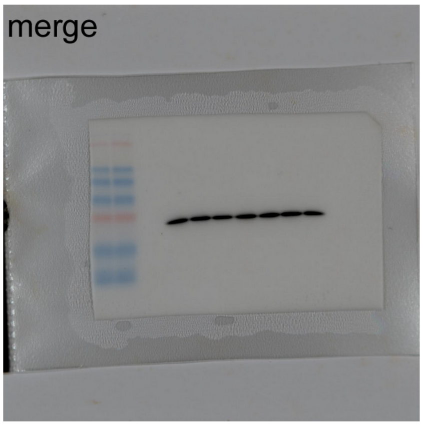

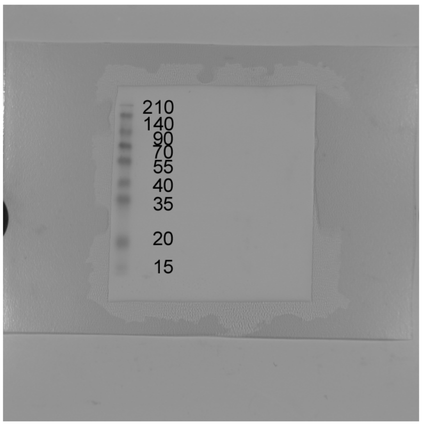

Figure 2a 24h GAPDH

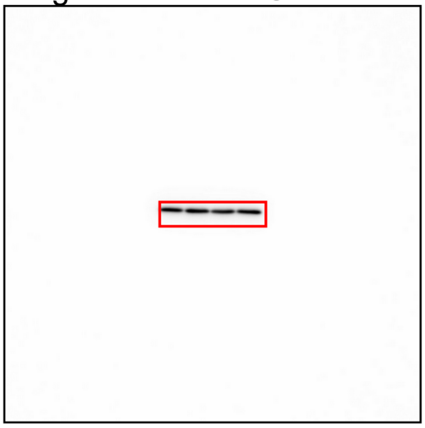

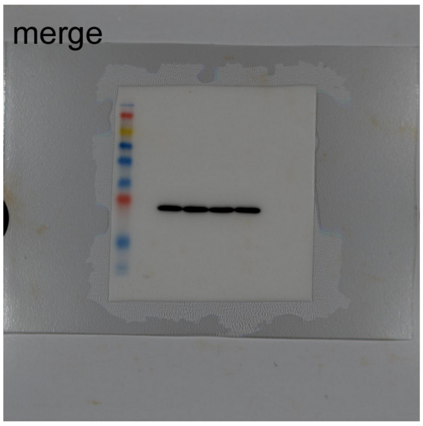

Original blot 4

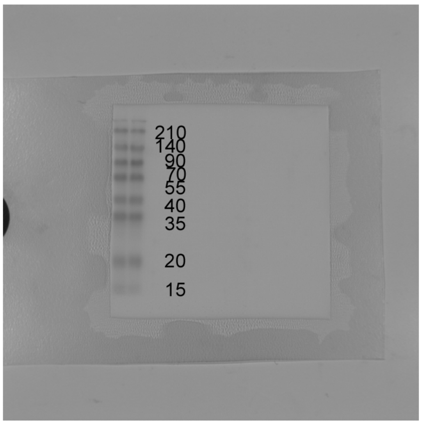

Figure 2a 48h GAPDH

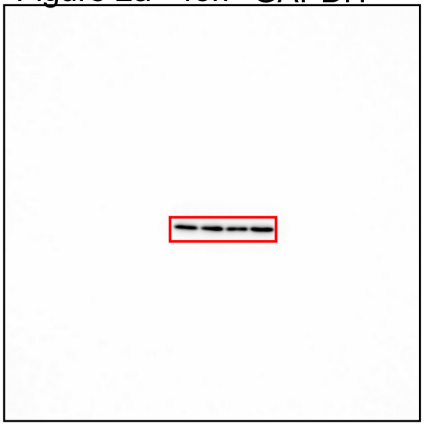

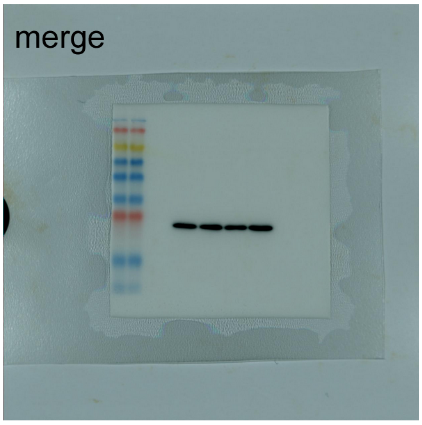

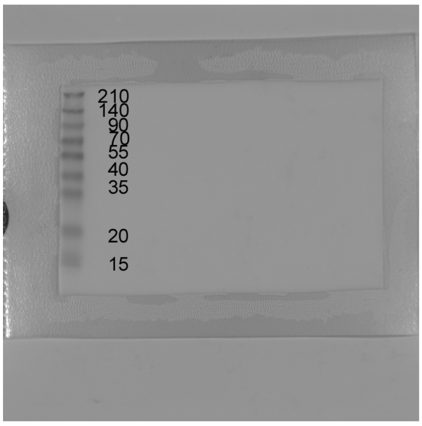

Figure 2c input PSMA7

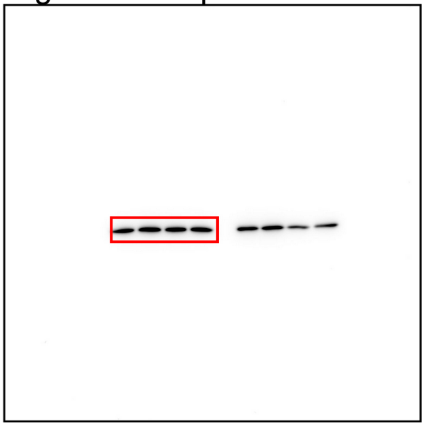

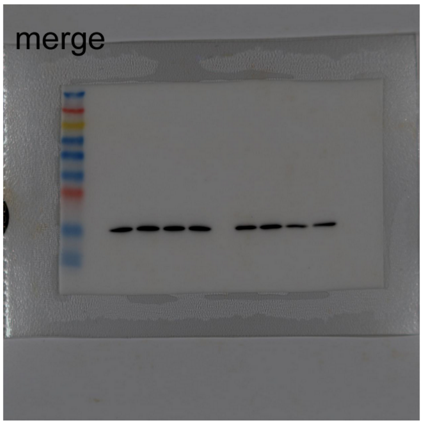

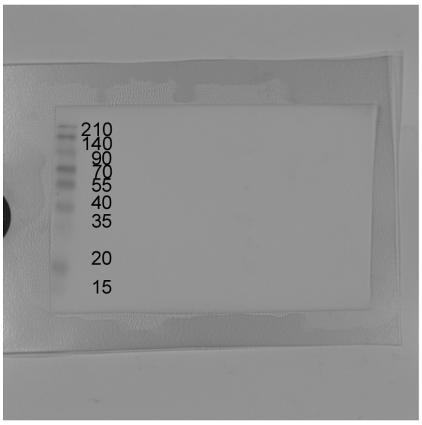

Figure 2c UbL-resin PSMA7

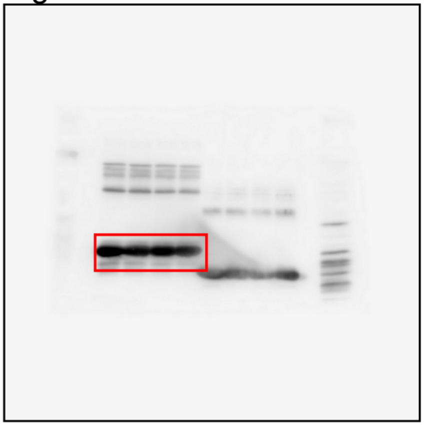

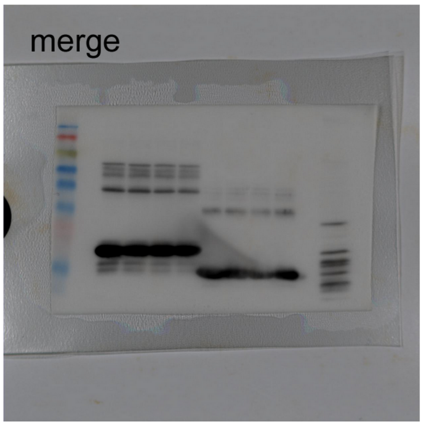

Original blot 6

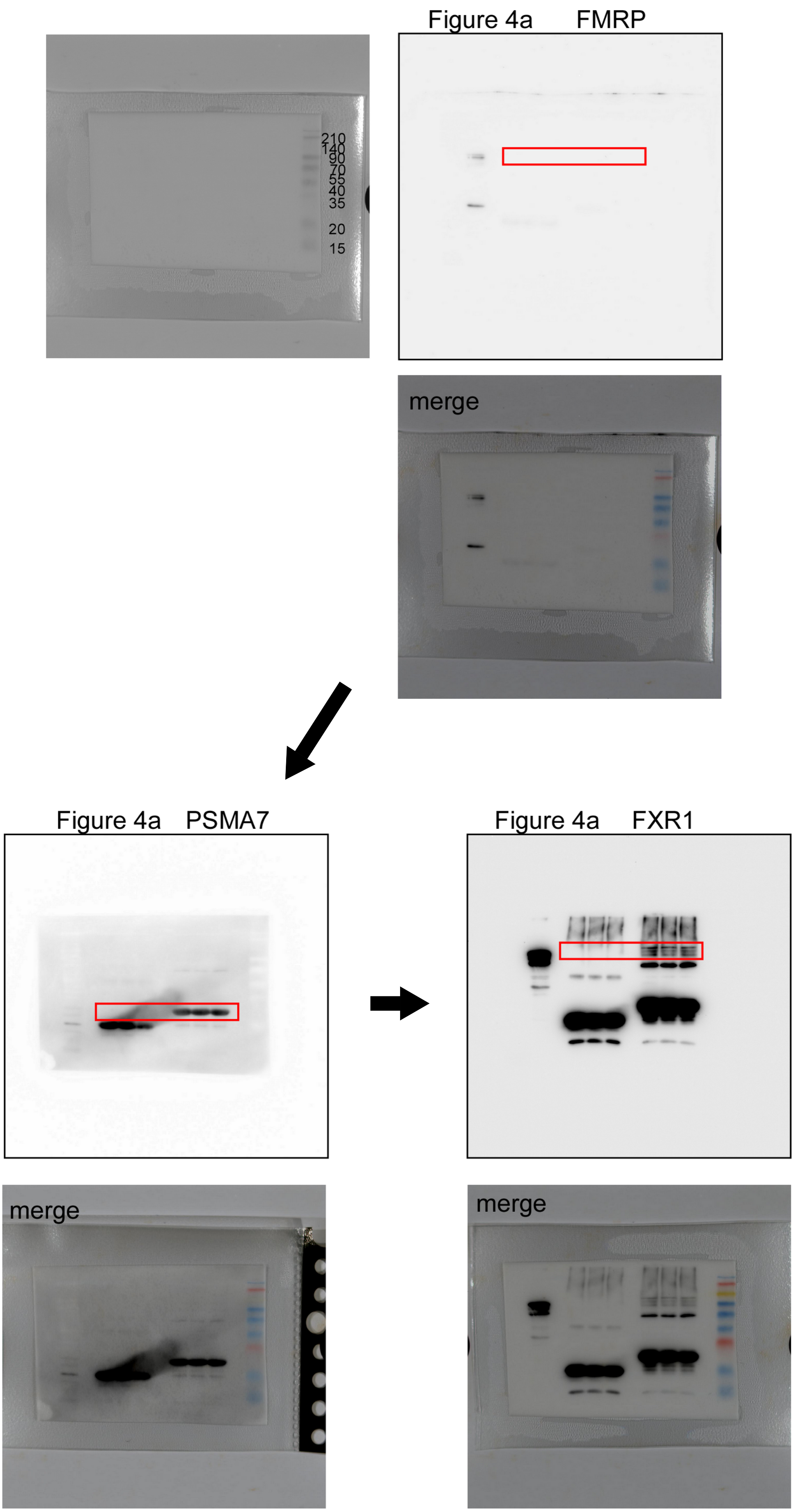

Original blot 7

UbL-resin

Figure 4b Control-resin FXR1

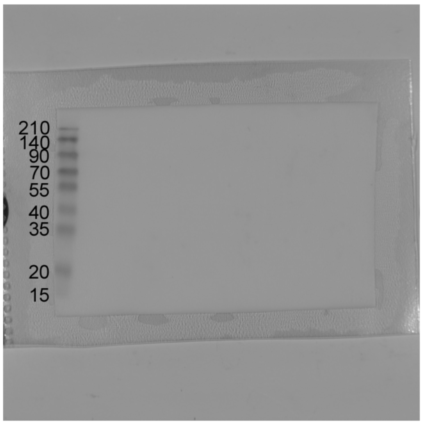

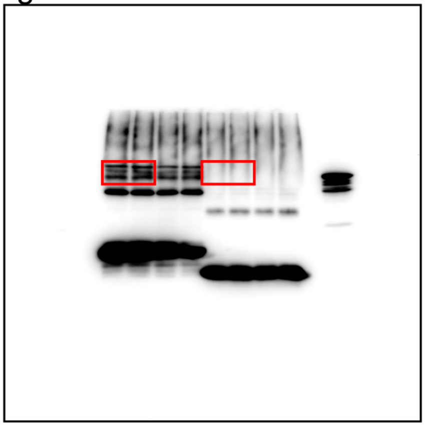

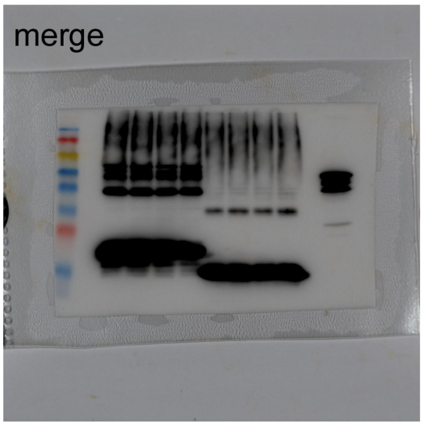

Figure 6b DLG4

Figure 6b βIII-tubulin

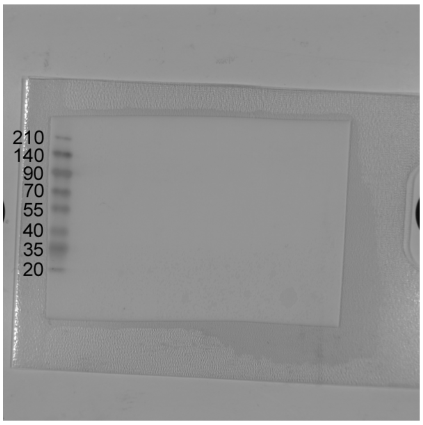

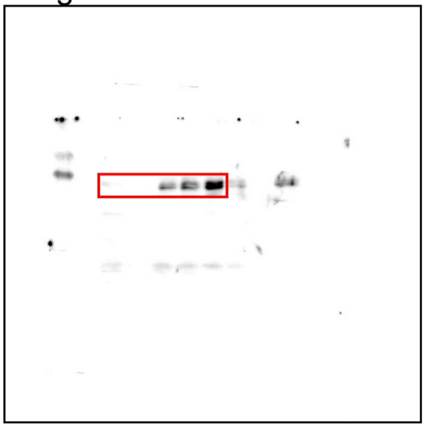

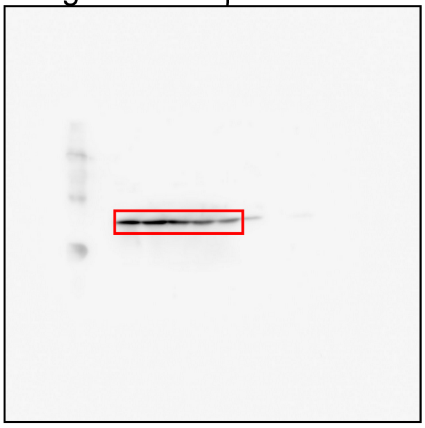

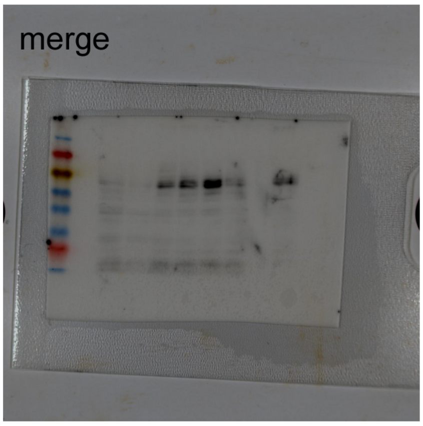

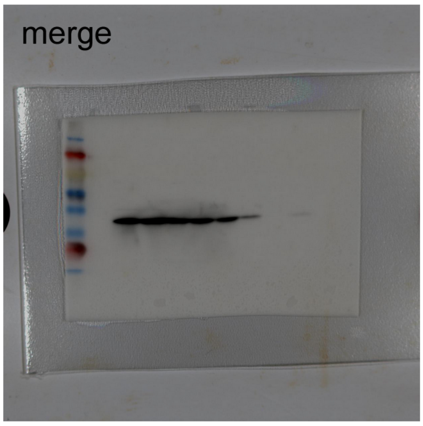

Original blot 8

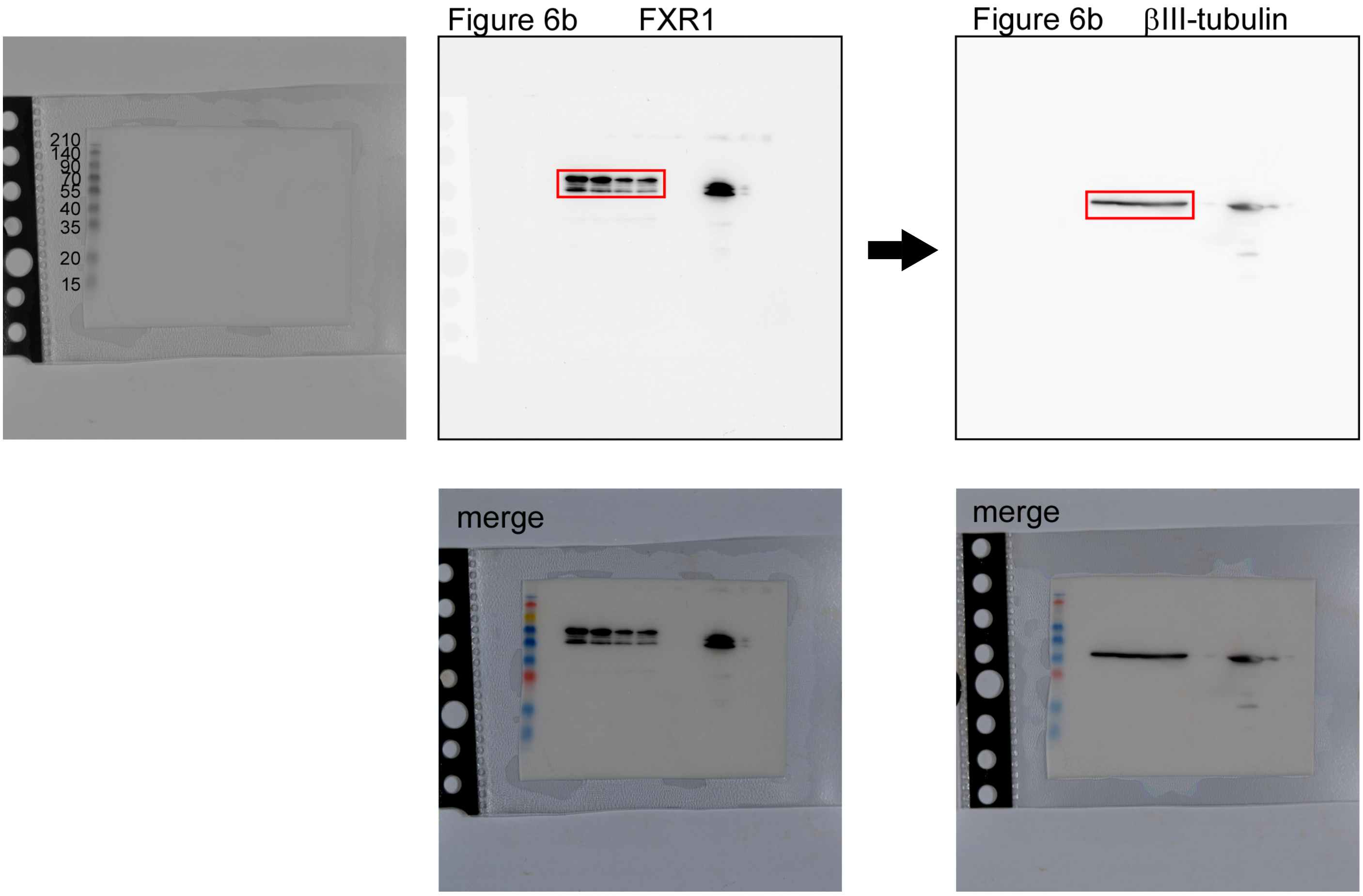

Original blot 9

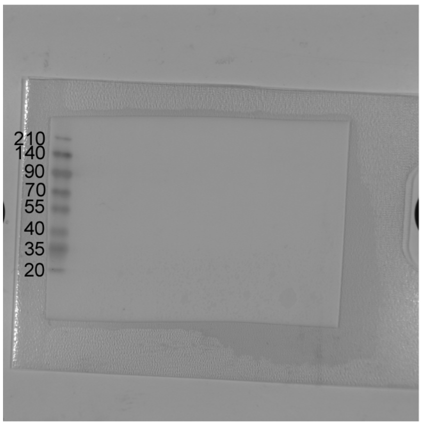

Figure 6c DLG4

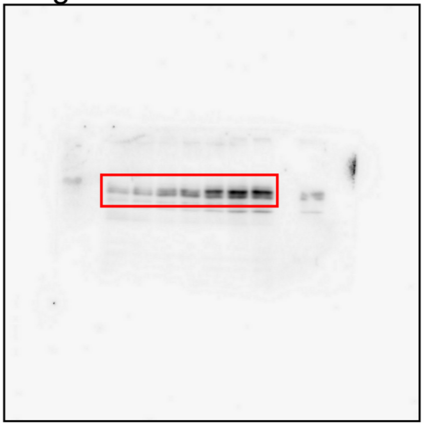

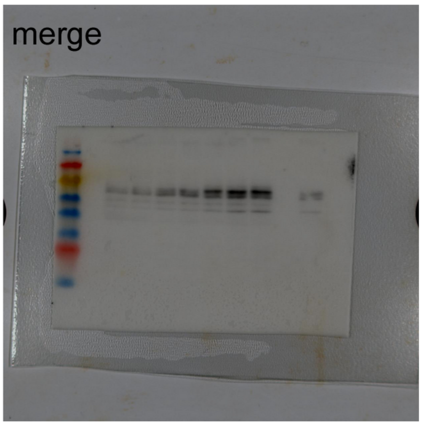

Figure 6c FXR1

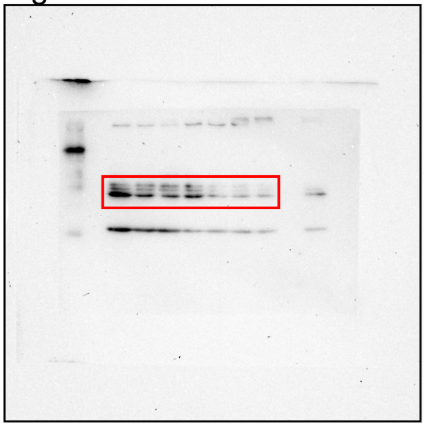

Figure 6c ACTIN

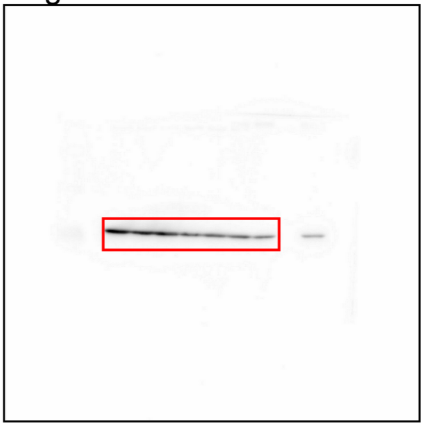

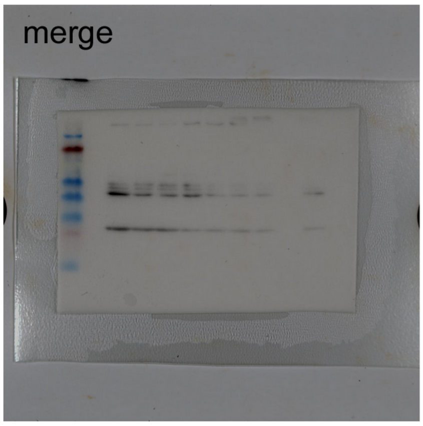

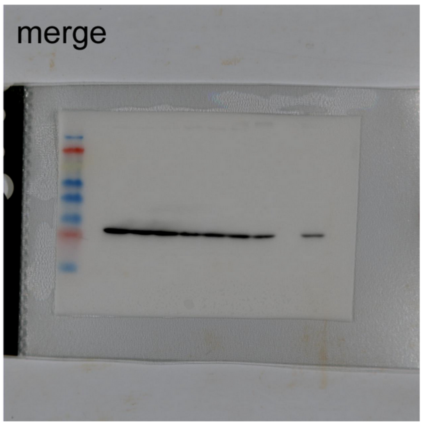

Original blot 10

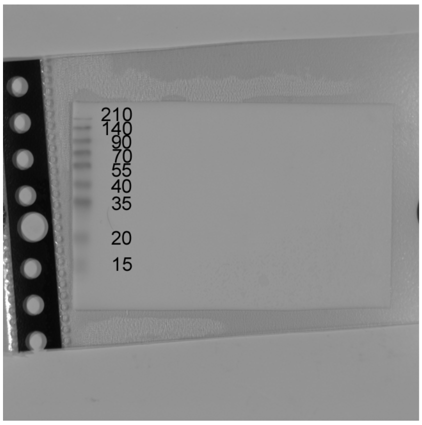

Suppl. fig 1 Exp.2 GFP

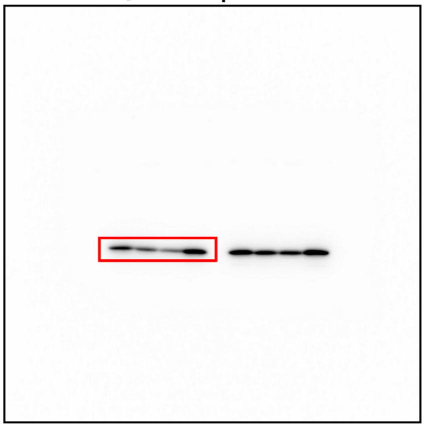

Suppl. fig 1 Exp.2 GAPDH

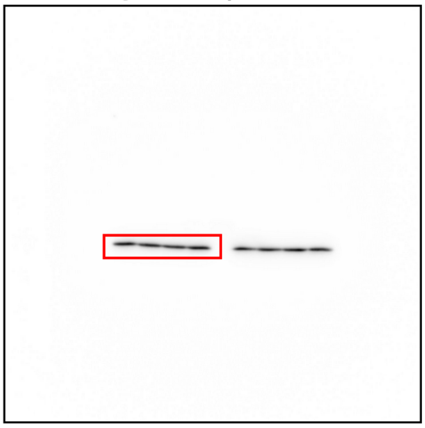

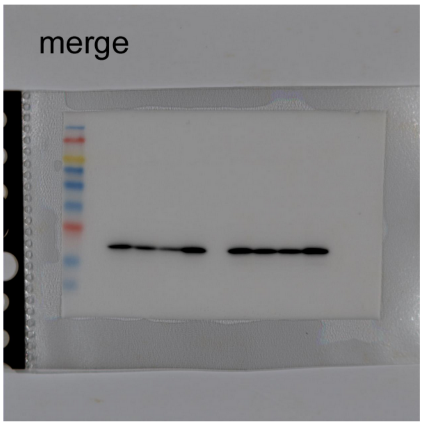

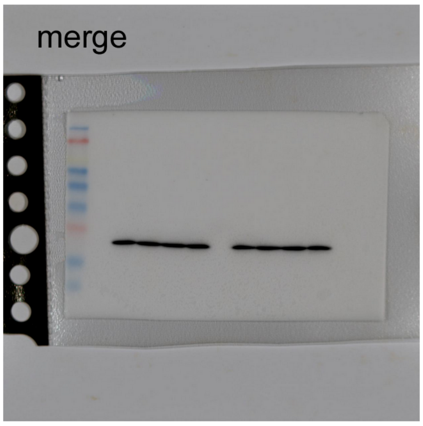

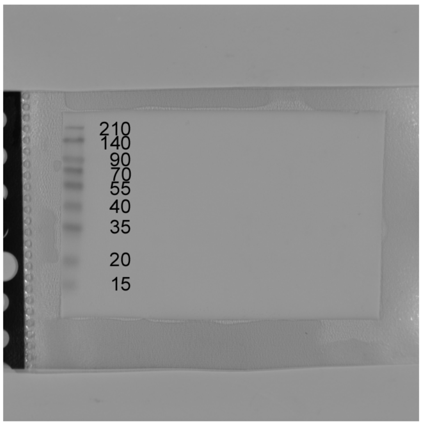

Suppl. fig 1 Exp.3 GFP

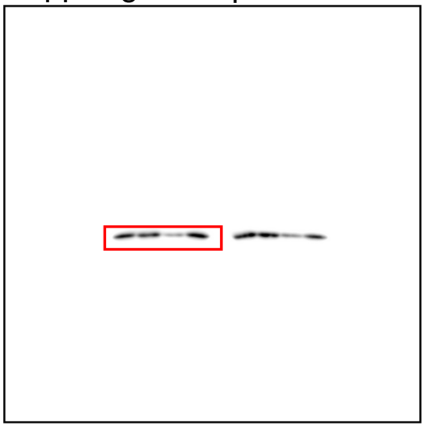

Suppl. fig 1 Exp.3 GAPDH

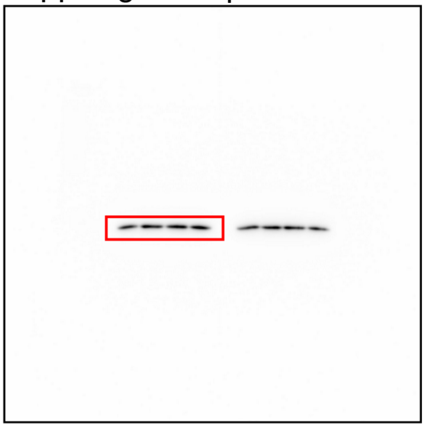

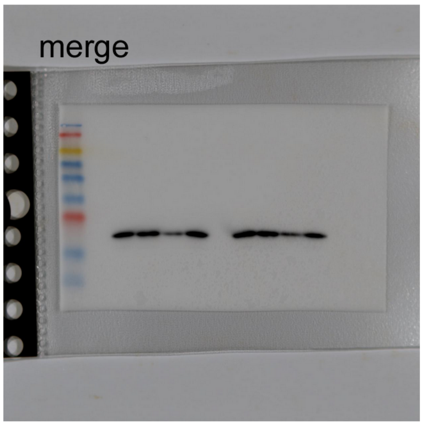

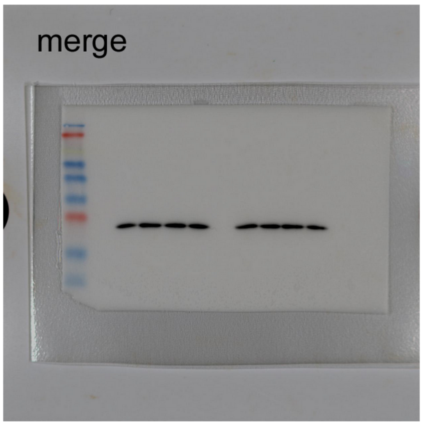

Original blot 11

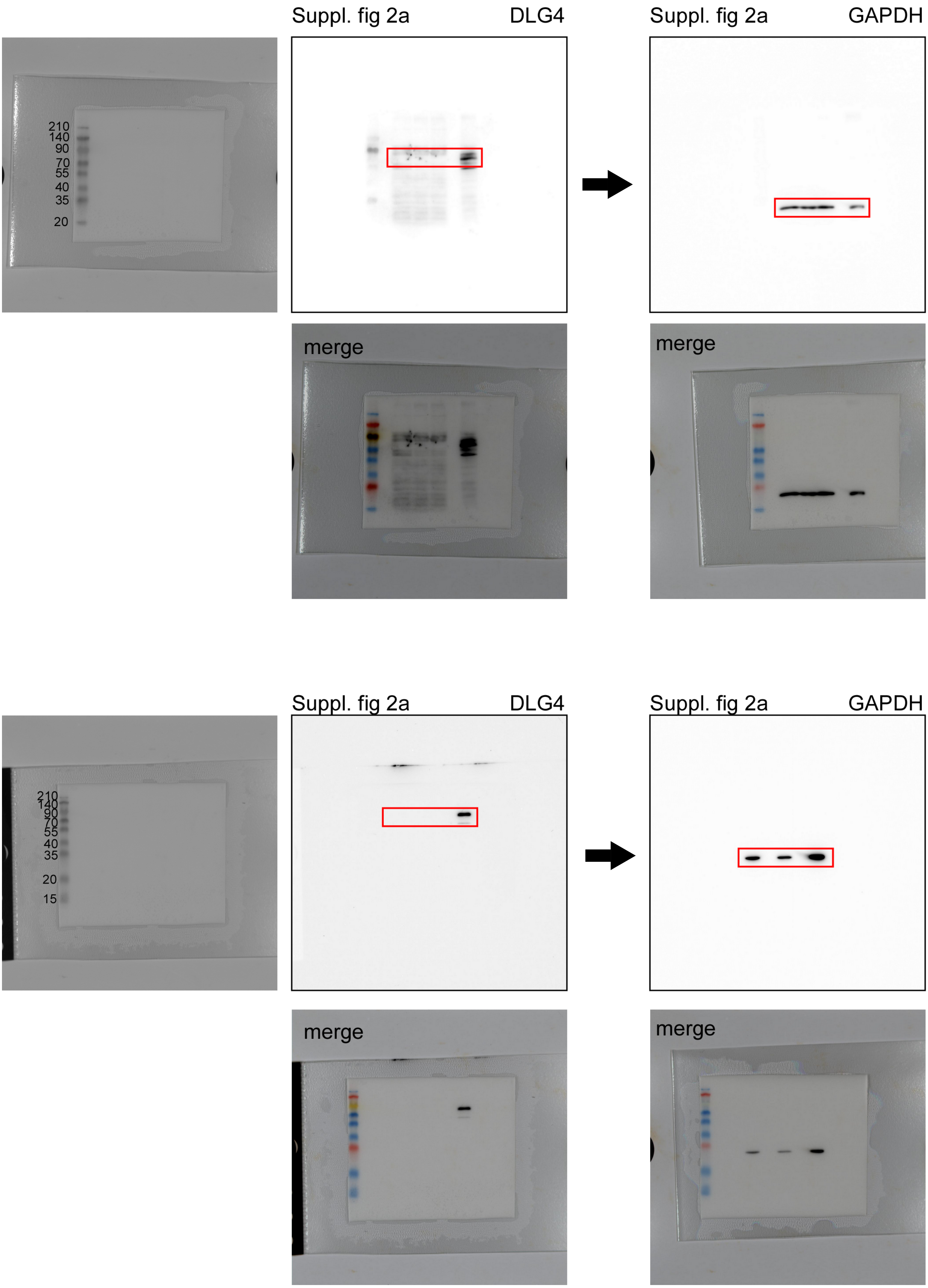

Original blot 12

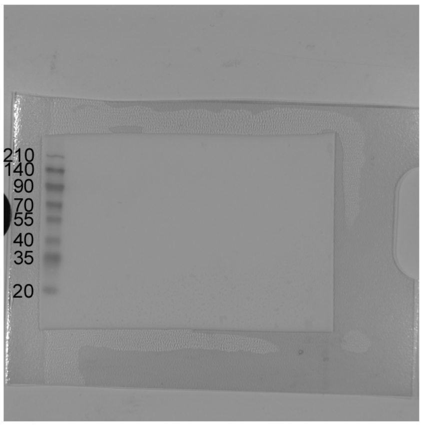

Suppl. fig 2b FXR1

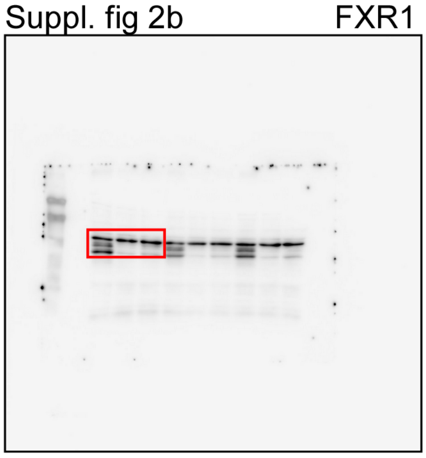

Suppl. fig 2b GAPDH

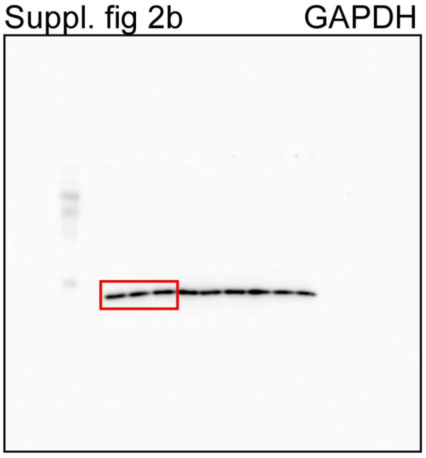

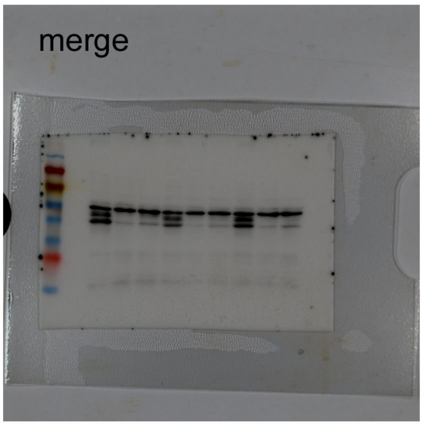

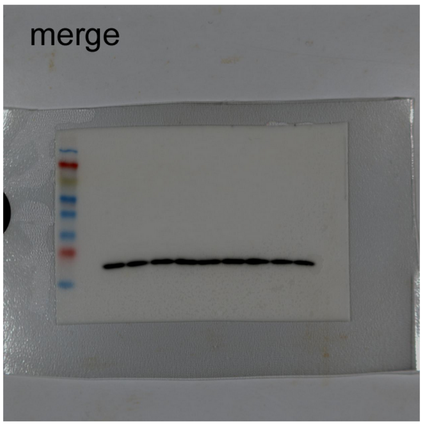

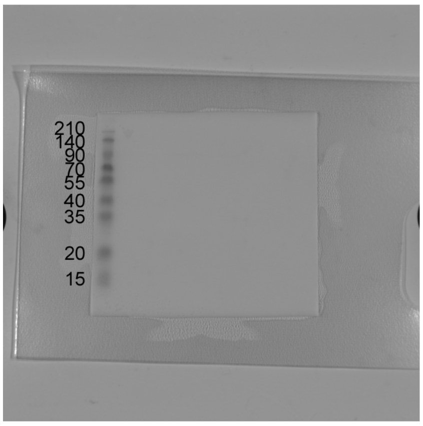

Suppl. fig 2c FMRP

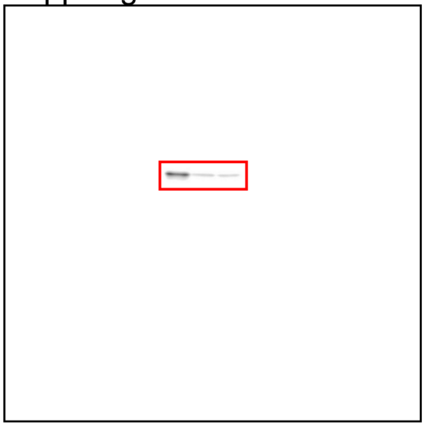

Suppl. fig 2c GAPDH

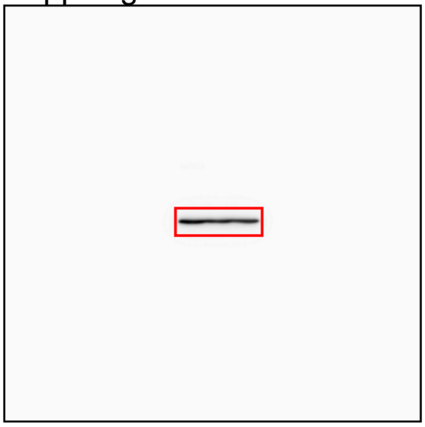

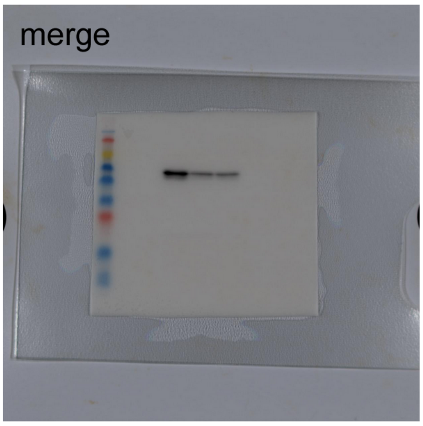

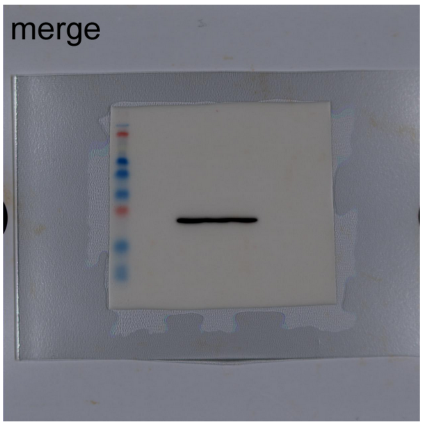

Original blot 13

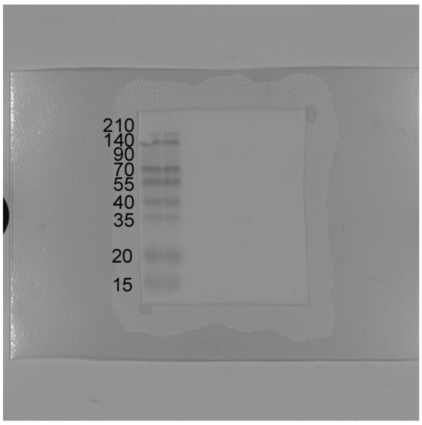

Suppl. fig 2d PRNP

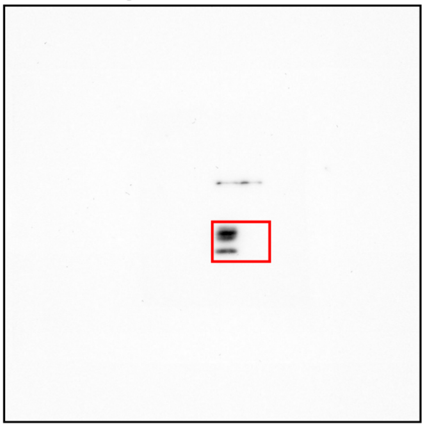

Suppl. fig 2d GAPDH

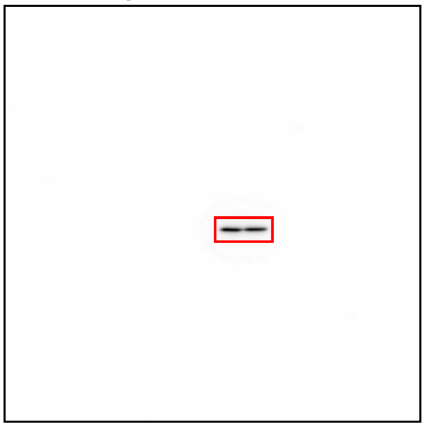

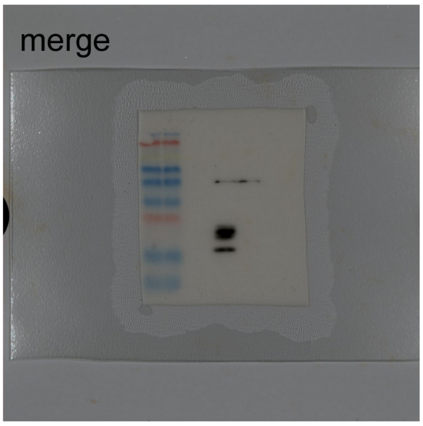

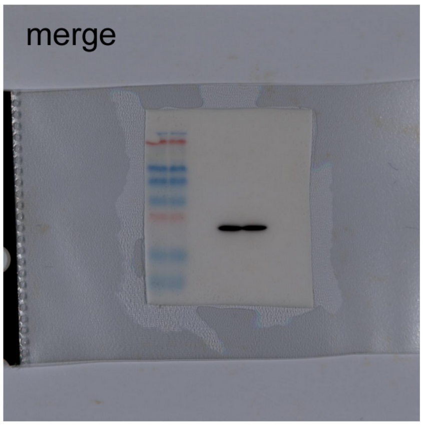

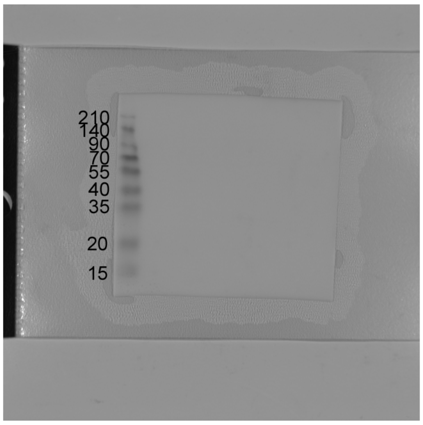

Suppl. fig 2d ATF4

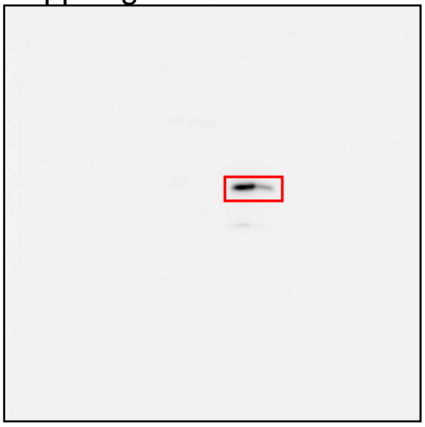

Suppl. fig 2d GAPDH

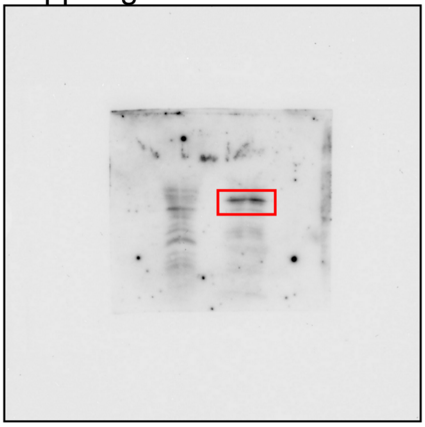

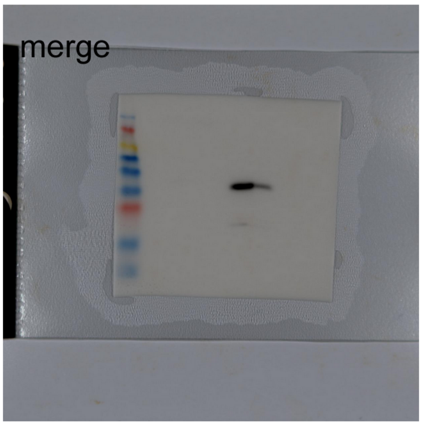

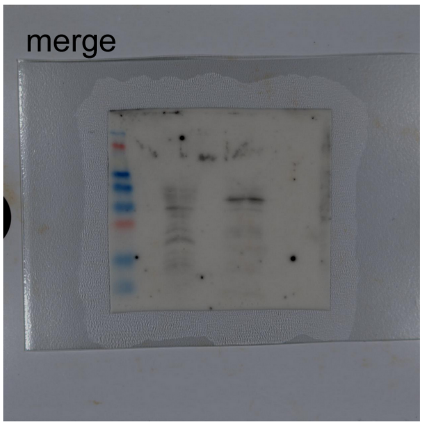

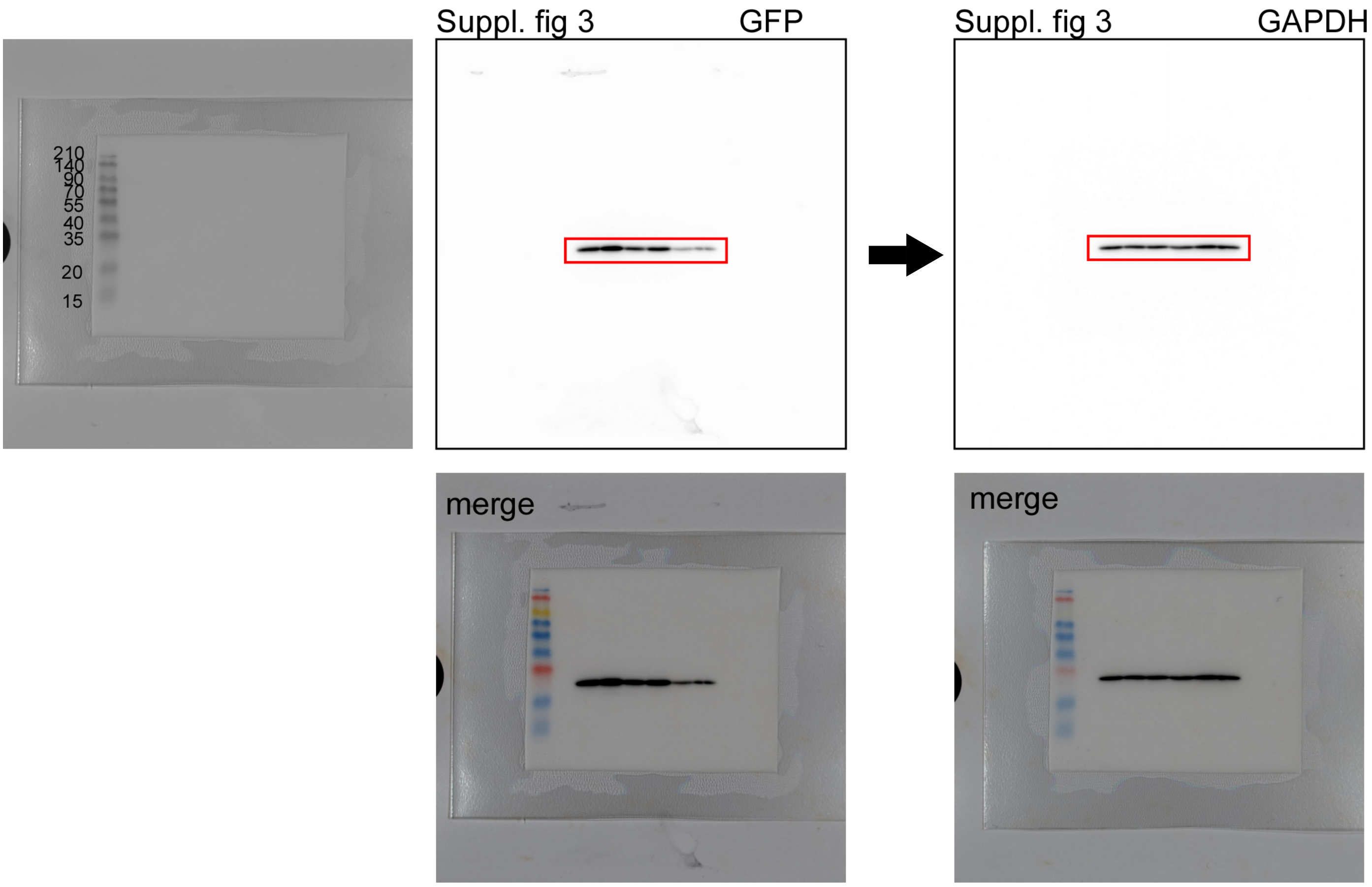
210
140
90
70
55
40
35
20
15
Suppl. fig 3
GFP
Suppl. fig 3
GAPDH
merge
merge

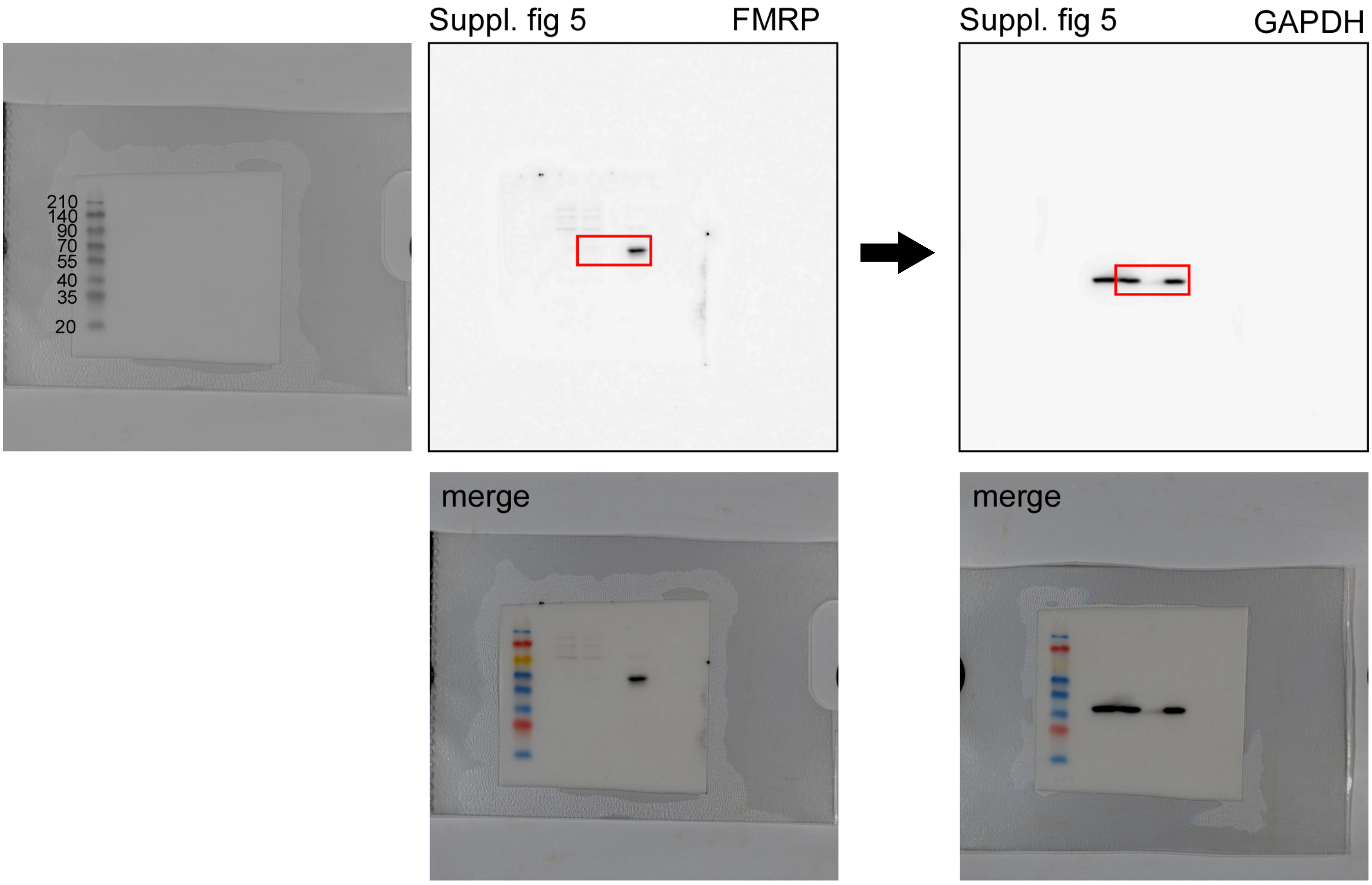
210
140
90
70
55
40
35
20
Suppl. fig 5
FMRP
Suppl. fig 5
GAPDH
merge
merge
